# Multidirectional in silico and in vitro Research for the Pharmaceutical Potential of Fibigia Clypeata (L.) Medik: Phytochemical, Antimicrobial, and Antimyeloma Properties

**DOI:** 10.1002/open.202500036

**Published:** 2025-09-04

**Authors:** Tuba Unver, Ugur Uzuner, Dilara Akcora‐Yildiz, Ismet Gurhan, Caglar Arkan, Zeynep Ozdemir

**Affiliations:** ^1^ Department of Pharmaceutical Microbiology Faculty of Pharmacy Inonu University 44280 Malatya Turkiye; ^2^ Department of Molecular Biology and Genetics Faculty of Science Karadeniz Technical University 61080 Trabzon Turkiye; ^3^ Department of Biology Faculty of Science & Art Mehmet Akif Ersoy University 15200 Burdur Turkiye; ^4^ Department of Pharmaceutical Botany Faculty of Pharmacy Inonu University 44280 Malatya Turkiye; ^5^ Department of Pharmaceutical Chemistry Faculty of Pharmacy Inonu University 44280 Malatya Turkiye

**Keywords:** antimicrobial activity, antimyeloma activity, *Fibigia clypeata*, molecular docking, natural pharmaceuticals

## Abstract

*Fibigia clypeata* (L.) Medik, a member of the Brassicaceae, has been the subject of limited research on its pharmaceutical and medicinal properties. This study aims to evaluate the phytochemical, antimicrobial, and antimyeloma properties of *F. clypeata* extracts and detail these results in silico analyses. The minimum inhibitory concentration (MIC) of *F. clypeata* extracts was determined using dilution methods, and antimyeloma activity was determined using an MTT (3‐(4,5‐dimethylthiazol‐2‐yl)−2,5‐diphenyl‐2H‐tetrazolium bromide) assay. The findings were evaluated by in silico analyses and correlated with the results of liquid chromatography‐high‐resolution mass spectrometry. The inhibitory effect of the water extract (MIC is 15 mg mL^‐1^ against bacterial strains; MICs are between 7.5 and 3.75 mg mL^‐1^ against *Candida* strains) was determined to be more potent than methanol extract (MIC is 60 mg mL^−1^ against bacterial strains; MICs are between 30 mg/mL and 7.50 mg mL^−1^ against *Candida* strains). Molecular docking findings revealed that cyanidin 3‐rutinoside chloride showed the highest binding affinity to *Staphylococcus aureus* MurB, *Candida parapsilosis*, and *Candida albicans* dihydrofolate reductases and the antitumor target human epidermal growth factor receptor protein. Based on MTT results, *F. clypeata* extracts significantly decreased cell viability dose‐dependently in three human MM and noncancerous MCF10A cell lines. *F. clypeata* harbor valuable antimicrobial and moderately anticancerogenic compounds.

## Graphical abstract

*Fibigia clypeata* water extract has a slightly more substantial inhibitory effect than the methanol extract. *F. clypeata* decreased cell viability in three human MM cells and the MCF10A cell lines. Cyanidin 3‐rutinoside chloride has the highest binding affinity to *Staphylococcus aureus* MurB, *Candida parapsilosis*, and *Candida albicans* dihydrofolate reductases and the antitumor target human epidermal growth factor receptor.

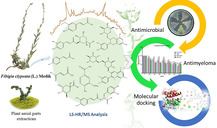

## 1 Introduction

Humanity's tendency towards more natural pharmaceuticals other than chemical therapeutic agents has created the need to study plants in more detail. Our ancestors used plants to treat many symptoms that threaten human health. Secondary metabolites of plants are biologically active chemical compounds that show primary effectiveness in pharmaceutical treatment. Phenolic compounds are secondary metabolites naturally found in plants with biological effects such as antidiabetic, anticancer, and antioxidant properties.^[^ ^1^, ^2^, ^3^, ^4^, ^5^ ^–^ ^6^ ^]^ Phenolic compounds are integral to human food and plant metabolites found naturally in plant products, propolis, and almost all plant materials.^[^ ^7^ ^]^ Some studies have reported that these biologically active compounds are antimicrobial, antiatherogenic, anti‐inflammatory, anticarcinogenic, and antiviral.^[^ ^8^, ^9^ ^]^ Phenolic compounds are widely used as antimicrobial preservatives in cosmetics, pharmaceuticals, beverages, and foods. In studies of the potential toxicities and pharmacological actions of phenolic compounds, it has been found that each phenolic product has a low toxicity, which rises as the alkyl chain length increases.^[^ ^9^ ^]^ In this study, the phenolic acids present in the plant *F. clypeata*, whose pharmacological effects have not been sufficiently investigated in the literature, were identified. At the same time, it was discussed how much the phenolic acids contained in the extracts obtained from this plant contributed to the detected antimicrobial and antimyeloma properties.

Multiple myeloma (MM), characterized by the proliferation of abnormal plasma cells in the bone marrow, is promoted by enhanced unfaithful DNA repair and mitochondrial activity.^[^ ^10^, ^11^ ^]^ The clinical symptoms of MM are anemia, hypercalcemia, lytic bone lesions, and renal impairment, so patients with MM mostly present with renal failure, bone fractures, and hence significant morbidity. The disease progression of MM is mainly preceded by a precursor asymptomatic condition named monoclonal gammopathy of indefinite significance and then smoldering myeloma (SMM). The progression from SMM to MM is most likely through clonal evolution and the acquisition of additional genetic events.^[^ ^12^ ^]^ Despite the latest advances in MM treatment, including high‐dose chemotherapy, stem cell transplantation, and the usage of novel proteasome inhibitors such as bortezomib and carfilzomib as well as daratumumab monoclonal antibody, MM remains an incurable disease due to the rapid acquisition of resistance to the given antimyeloma agents.^[^ ^13^ ^]^ Therefore, there is an immediate unmet need for effective therapy for MM. Many herbal extracts are a natural source of bioactive compounds/drugs that exhibit antioxidant and antitumorigenic activities.^[^ ^14^ ^]^ However, the antimyeloma effect of *Fibigia clypeata* needs to be elucidated.

*F. clypeata* (L.) Medik is a species in the Brassicaceae family. It spreads naturally in almost all Anatolian regions except the Mediterranean region in Turkey.^[^ ^15^ ^]^ It grows between 500 and 2300 m altitudes on stony slopes where water is scarce. *F. clypeata* can grow up to 40 cm. It is woody at the base and densely gray‐haired, and its stem erect, unbranched, or branched. The plant's stem is sessile, and the flowers are yellow and densely clustered. *F. clypeata* fruit is elliptical and ciliated, and the seeds are arranged in two rows.^[^ ^15^, ^16^ ^]^ Although studies on the pharmacological activity of *F. clypeata* are very limited, one study reported that *F. clypeata* may control Alzheimer's disease, skin hyperpigmentation problems, and type 2 diabetes. It has been proven that methanol extract has higher antioxidant activity, and it has been stated that anticancer and advanced analysis studies are needed.^[^ ^8^ ^]^ In our study, for the first time, the in vitro antibacterial, antifungal, and antimyeloma properties of *F. clypeata* extracts were investigated, and this study was supported by in silico analyses.

## 2 Experimental Section

### 2.1 Plant Materials and Extractions

**Figure 1:**
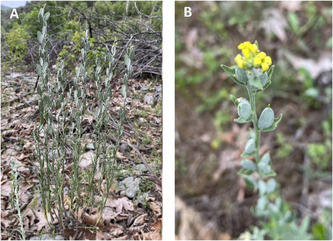
Pictures of *Fibigia clypeata* (L.) Medik. A) *F. clypeata* in its natural habitat. B) Flower of *F. clypeata*.

*F. clypeata* was collected from the Puturge‐Kurucay and Merap mountain slopes of Malatya province (Turkey) in mid‐May 2022 (**Figure** **1**). The plant was identified in the Pharmaceutical Botany Laboratory, Faculty of Pharmacy, Inonu University, and stocked in the Inonu University Faculty of Pharmacy Herbarium (Voucher Number: TU1002).^[^ ^15^ ^]^ Firstly, the plant aerial parts, including flowers, stems, and leafs, were dried, crushed by a grinder, and extracted. For extraction performance, methanol and water were used as solvents. Ten grams of the plant aerial parts were added to 100 mL of water and methanol separately and left to macerate at room temperature (15–20 °C) for 24 h. The filtrate was then removed, and the solvent was added. This procedure was repeated four times (4 × 24 h). The collected filtrates were combined and evaporated by a rotary evaporator (Heidolph Laborota 4000, Germany).^[^ ^17^ ^]^ The obtained extracts were stored in the refrigerator at −20 °C.

### 2.2 Liquid Chromatography‐High Resolution Mass Spectrometry (LC‐HR/MS) Analysis

The LC‐HR/MS analyses were carried out using a Reversed Phase C18 column on an Agilent 6460 Triple Quad LC‐MS/MS with 1290 Infinity UPLC system and an Electrospray Ionization (ESI) Source. The mobile phases A and B comprised 0.1% formic acid in water and 0.1% in acetonitrile, respectively. An injection volume of 5 μL was loaded onto a Zorbax SB‐Aq (1.8 μm particle size, 2.1 mm internal diameter × 50 mm length) analytical column held at 30 °C and equipped with a Zorbax SB‐C8 Rapid Resolution Cartridge (3.5 μm, 2.1 × 30 mm) guard column, also from Agilent Technologies. The gradient elution was applied to mobile phase B at concentrations ranging from 5 to 5‐20‐90‐90‐5‐5 after 0‐4‐7‐14‐15−15.1‐20 min, with a flow rate of 0.4 mL min^−1^.^[^ ^18^ ^]^ The samples were determined by comparing the picks of extracts and retention durations of reference compounds with HR/MS data from the Eastern Anatolia High Technology Application and Research Center (DAYTAM, Erzurum, Turkey). One milligram of dry water and methanol extract was dissolved in the mobile phase (1 mL; A: B; 50:50; v/v). Before being injected into the LC‐MS/MS in a volume of 5 µL, the solution was filtered via a 0.45‐m filter. The mass spectrometer was operated in both positive (+ESI) and negative (‐ESI) ionization modes. The ESI source settings were as follows: nebulizer pressure 45 psi; desolvation gas flow rate 11 L min^−1^ at 325 °C; source temperature and gas flow rate, 200 °C and 14 L min^−1^, respectively; capillary voltage 4 kV; and fragmentor 140 V. Data were acquired using MassHunter Data Acquisition (Agilent Technologies, version 6.0) over the 100–1200 m/z range at a rate of 1.5 spectra/s. Molecules were tentatively identified by comparison of the exact mass with published data.^[^ ^18^ ^]^

### 2.3 Antimicrobial Activity

#### 2.3.1 Strains and Media

Nine microorganisms (5 bacteria and 4 yeast), including *Enterobacter aerogenes* (ATCC 13048), *Staphylococcus aureus* (ATCC 12600), *Klebsiella pneumoniae* (ATCC 13883), *Escherichia coli* (ATCC 10536), *Pseudomonas aeruginosa* (ATCC 10145), *Candida tropicalis* (ATCC 13803), *Candida albicans* (ATCC 14053), *Candida krusei* (ATCC 14243), and *Candida parapsilosis* (ATCC 22019) were used for the antibacterial and antifungal activity assay. All strains were obtained from the American Type Culture Collection (ATCC). Muller Hinton Broth (Himedia, Nashik, India) and Muller Hinton Agar (Merk, Darmstadt, Germany) for bacterial strains and Sabouraud Dextrose Broth (Biolife, Milano, Italy) and Sabouraud 4% glucose agar (Chemsolute, Renningen, Germany) for yeast strains were used for *F. clypeata* extracts antimicrobial activity assay.

#### 2.3.2 Agar Dilution Method

The agar twofold dilution method was carried out for the antimicrobial activity of *F. clypeata* extracts, and the previous protocol was performed with minor modifications.^[^ ^19^, ^20^ ^]^ The first agar plates had a concentration of 720 mg/6 mL (w/v) of *F. clypeata* methanol and water extracts, and a twofold dilution was performed. Therefore, the concentration of the extracts in the agar plates from the A plate to the K plate ranged from 120 to 0.12 mg/mL (w/v). As control plates, pure Muller Hinton agar for bacterial strains and Sabouraud glucose agar for yeast strains were prepared separately. Standard inoculum was prepared for each species in distilled water, and their turbidity was set to 0.5 McFarland standard (1 × 10^8^ cfu/mL for bacterial strains, 1 × 10^6 ^cfu/mL for *Candida*). Subsequently, Muller Hinton agar plates were divided into five sections for bacterial strains (*S. aureus* to Section 1, x*E. aerogenes* to Section 2, *E. coli* to Section 3, *P. aeruginosa* to Section 4, and *K. pneumoniae* to Section 5). Furthermore, Sabouraud glucose agar plates were divided into four sections for yeast strains (*C. albicans* to section 1, *C. tropicalis* to Section 2, *C. krusei* to Section 3, and *C. parapsilosis* to Section 4). One microliter of each microorganism from the standard inoculum was inoculated onto the agar plate surface using an inoculation loop, in which different *F. clypeata* extract concentrations were included. Then, plates were incubated at 36 °C for 24 h. Afterward, the minimum inhibitory concentration (MIC) values were determined based on the apparent growth of each microorganism on the plates (Supporting Information‐SI 1). Each experiment was performed, including triplicate treatments.

#### 2.3.3 Broth Dilution Method

The broth twofold microdilution method was used in the antibacterial and antifungal assay of *F. clypeata* water and methanol extracts, and the CLSI standard methodology was performed with minor modifications.^[^ ^19^, ^20^ ^]^ The antimicrobial activity tests of *F. clypeata* extracts were replicated against nine microbial strains using a 96‐well microplate. Initially, a twofold dilution was made by adding 24 mg of *F. clypeata* extracts into 200 µl of Muller Hinton Broth and 200 µl of Sabouraud Dextrose Broth for bacterial and yeast strains, respectively. As a result, extract concentrations from 120 to 0.12 (mg  mL^−1^) were added to wells 1st to 10th. Pure Muller Hinton Broth and Sabouraud Dextrose Broth were placed in the control wells for negative control to confirm the nonexistence of contamination. Microorganisms used within Muller Hinton Broth and Sabouraud Dextrose Broth were placed in the other well for positive control to confirm microorganism viability. The standard inoculum of each strain was prepared in distilled water, and their turbidity was set to 0.5 McFarland. After that, 1 µl of each microorganism was inoculated from the A row to the I row. The microplate, which includes different extract concentrations of *F. clypeata*, was incubated at 36 °C for 24 h. Then, 15 µl of resazurin (0.15% w/v) (CAS No:62758‐13−8, Sigma–Aldrich, USA) was added to each well. After it was incubated at 36 °C for 4–6 h, the growth of bacterial and yeast strains was assessed by observing the color change.

### 2.4 Determination of the Antimyeloma Effect of *F. clypeata* by MTT Assay

Three human MM cell lines, NCI H929, RPMI 8226, U266, and noncancerous mammary gland epithelial cell line MCF10, were used to explore the cytotoxicity of *F. clypeata* by MTT (3‐(4,5‐dimethylthiazol‐2‐yl)−2,5‐diphenyl‐2H‐tetrazolium bromide) (Sigma–Aldrich, St Louis, MO, USA) assay according to the manufacturer's instructions. MM cell lines and MCF10A were cultured in RPMI 1640 and DMEM, respectively, supplemented with 1% penicillin‐streptomycin and 10% heat‐inactivated fetal bovine serum in a 37 °C incubator with 5% CO2 humidification. MM and MCF10A cells were cultured with various concentrations of *F. clypeata* or without for 48 h in 96‐well plates at a density of 40,000 and 10,000 cells per well, respectively. After treatment with various concentrations of *F. clypeata* methanol and water extracts, 5 mg mL^−1^ of the MTT reagent was added to each well and then incubated at 37 °C for 2.5 h, followed by DMSO treatment and incubation in the dark at 37 °C for overnight. Next, the absorbances were read at the BioTek Epoch Microplate Spectrophotometer (Agilent Technologies). Untreated cells used as control were treated with methanol or water in the culture media at an equal volume of the highest concentration. The percentage of cell viability was calculated relative to untreated cells, which was considered 100%. The MTT results were obtained from three independent replications of three wells as intra‐experimental repeats.

## 3 In silico‐Molecular Docking

Multifaceted molecular docking studies were performed to examine the antimycotic and anticancer effects of *F. clypeata* natural compounds. To investigate the antibacterial activity potential of *F. clypeata* natural compounds, *S. aureus* UDP‐N‐acetylenepyruvylglucosamine reductase (MurB; PDB ID: 1HSK; Resolution: 2.30 Å) enzyme was targeted for molecular docking studies. MurB is an enzyme required for bacterial peptidoglycan biosynthesis and is an alluring target for antimicrobial drug development. MurB contains noncovalently bound flavin adenine dinucleotide (FAD) as a cofactor. It catalyzes the NADPH‐dependent reduction of UDP‐N‐acetyleneolpyruvylglucosamine (UDP‐GlcNAcEP) to UDP‐N‐acetylmuramic acid. The reduced product then serves as the binding site for the peptide portion of the cell wall.^[^ ^21^ ^]^

In silico molecular docking studies on both *C. parapsilosis* and *C. albicans* dihydrofolate reductase (DHFR; PDB ID: 4HOE; Resolution: 1.76 Å) were performed to investigate the antifungal properties of *F. clypeata* natural compounds. Dihydrofolate reductase (DHFR) in fungi is required for the reduction reaction of dihydrofolate to tetrahydrofolate (THF). It uses nicotinamide adenine dinucleotide phosphate (NADPH) as the electron donor for this catalytic reaction. THF is essential for the action of folate‐dependent enzymes and is required for synthesizing purines and thymidylates, which are important for DNA replication and methylation. DHFR is, therefore, vital for synthesizing nucleic acid precursors, cell proliferation, and cell growth.^[^ ^22^ ^]^ The DHFR target was evaluated as a promising drug target for studying the antifungal potential of *F. clypeata* natural compounds. Natural compounds of *F. clypeata* were also investigated for potential anticancer targets. For this, each small molecule was used for in silico target prediction analysis. *F. clypeata* compounds were specifically examined for their potential targets for myeloma tumors.

Molecular docking studies were conducted using the AutoDock Vina 1.5.7 program.^[^ ^23^ ^]^ The three‐dimensional (3D) structure of *C. albicans* DHFR (4HOE) was obtained from the Protein Data Bank.^[^ ^24^ ^]^ However, for *C. parapsilosis* DHFR, whose 3D structure has not yet been elucidated, the corresponding DHFR amino acid sequence for the functional domain was obtained from NCBI, and AlphaFold2‐based 3D structure prediction was then performed prior to molecular docking studies.^[^ ^25^ ^]^ The model quality of *C. parapsilosis* DHFR was validated by analyzing its protein structural coordinates through the PROCHECK program.^[^ ^26^ ^]^

Epidermal growth factor receptor (EGFR) upregulation was reported for several MM cancers.^[^ ^27^, ^28^ ^]^ We have also examined the antitumorigenic activities of *F. clypeata* compounds by analyzing their binding affinities to EGFR (PDB ID: 5UG9; Resolution: 1.33 Å). In addition, EGFR signaling has recently been proposed as a potential target for anti‐angiogenic therapy and MM.^[^ ^29^ ^]^

For molecular docking studies, bound ligands and water molecules were excluded from the target protein 3D structures. However, NADPH ligands bound to the active sites of DHFR enzymes were retained. Both target protein structures were energetically minimized, and their protonation states were counted with PROPKA 3.0 at pH = 7.0.^[^ ^30^ ^]^ The pdb files of the natural compounds were generated using Open Babel.^[^ ^31^ ^]^ The pdbqt files of extracted compounds and target proteins were created using AutoDock tools 1.5.7. Polar hydrogens were added to the 3D structures of DHFR and EGFR targets, and Kollman charges were also computed. For natural ligands, Gasteiger charges were calculated, and pdbqt files were created. During antifungal assays, the DHFR 3D structures of both *C. parapsilosis* and *C. albicans* were targeted to elucidate the different binding affinity of natural compounds to both targets.

Each docking experiment was performed on DHFRs related to antifungal activity and EGFR targets related to antitumor activity through three separate runs using native compound pdbqt files. The grid dimensions and grid center coordinates used during the molecular docking studies for each target were summarized in **Table** **1**. During the molecular docking studies on the EGFR target, the original ligand of 5UG9, which is 8 AM, was used as the control ligand. The docking scores for each compound were obtained, and specific 2D interaction maps were examined using the Discovery Studio Accelrys (Discovery Studio Visualizer‐ v21.1.0.20298) program (Dassault Systems, BIOVIA).

**Table 1:** Grid center coordinates and grid box dimensions for each protein targeted in molecular docking studies.

	Grid center coordinates in Å			Grid dimensions in Å		
Target protein	X	Y	Z	X	Y	Z
1HSK	−178	155	155	−30	25	30
Cparapsila)	−4	8	33	25	25	25
4HOE	−4	8	33	25	25	25
5UG9	−13	18	−26	25	30	25

a) Cparapsil: The best protein model of *C. parapsilosis* DHFR was obtained by executing AlphaFold2.

## 4 Results and Discussion

### 4.1 LC‐HR/MS Analysis of the Extracts

The LC‐HR/MS analysis of *F. clypeata* followed the spectrometric investigation. Of the 35 phenolic compounds analyzed via LC‐HR/MS, 26 were absent, whereas 9 were identified in methanol and water extracts. **Table** **2** lists the amount of each chemical, retention time (Rt), and the found molecular ion. 8.04 g kg^‐1^ of individual phenolic compounds were found in the water extract of the *F. clypeata*, while 1.94 g kg^‐1^ of individual phenolic compounds were found in the methanol extract. Fumaric acid, with an amount of 3.66 g kg^‐1^, was the most frequent substance in the water extract, followed by ferulic acid, with an amount of 1.73 g kg^‐1^. The most frequent ingredient in the methanol extract was cyanidin‐3‐O‐glucoside, which had a 0.54 g kg^‐1^ amount.

**Table 2:** Phenolic content in terms of amount in methanol and water extracts.

Compounds	Found m/z (molecular ion)	Rt (min)	Amount of phenolic comp. [g kg^−1^]	
			Water extract	Methanol extract
Quinic acid	190.9	2.477	0.0022	0.0560
Fumaric acid	114.9	4.032	3.6649	0.2454
Cyanidin 3‐rutinoside chloride	592.8	9.892	0.5546	0.2412
Cyanidin‐3‐*O*‐glucoside	447.1	10.372	0.3390	0.5435
Chlorogenic acid	352.9	10.824	–	0.0058
Vanilic acid	166.9	11.499	0.6885	0.3743
*p*‐Coumaric acid	163.0	12.312	0.9545	**–**
Rosmarinic Acid	358.8	12.462	0.1057	0.1279
Ferulic acid	193.0	12.542	1.7310	0.3448

### 4.2 Results of Antimicrobial Activity

#### 4.2.1 Antimicrobial Activity of *F. clypeata* Water Extract

**Figure 2:**
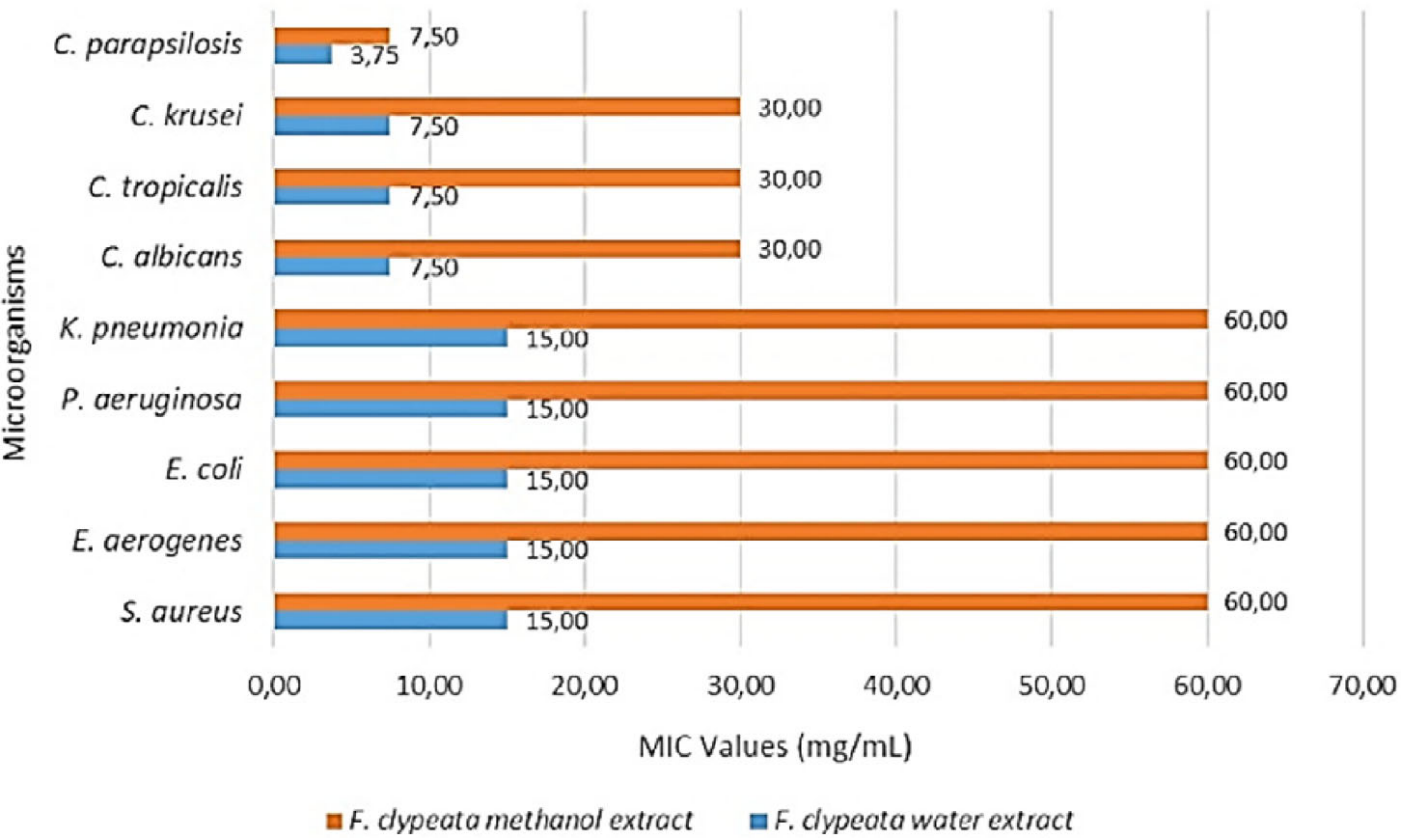
MIC values of *F. clypeata* water and methanol extracts against different microorganisms.

The antimicrobial activity results of *F. clypeata* water extract against bacterial strains using the agar dilution assay are demonstrated in SI 1. After 24 hr of incubation, plates were treated with 7.5 (plate E) to 0.12 mg mL^−1^ (plate L) of *F. clypeata* water extract, and the control plate was covered with *S. aureus* colonies. No colonies were determined on A, B, C, and D plates treated with 120, 60, 30, and 15 mg mL^−1^ of *F. clypeata* water extract, respectively. So, the MIC value of the water extract against *S. aureus* was determined to be 15 (mg mL^−1^). Like the inhibition effect of *F. clypeata* water extract against *S. aureus*, MIC values against other bacterial strains, including *E. aerogenes, P. aeruginosa, K. pneumonia*, and *E. coli* were determined as 15 mg mL^−1^ (**Figure** **2**).

The results of *F. clypeata* water extract antimicrobial activity against yeast strains using the agar dilution assay are demonstrated in SI 2. After 24 hr of incubation, colonies appeared from the F‐plate, where 3.75 mg mL^−1^ *F. clypeata* water extract concentration was used for *C. albicans, C. krusei*, and *C. tropicalis.* Thus, this study determined the MIC value of *F. clypeata* water extract against *C. albicans, C. tropicalis*, and *C. krusei* to be 7.5 mg mL^−1^. *C. parapsilosis* colonies began appearing from the G plate, where 1.87 mg mL^−1^ *F. clypeata* water extract concentration was used. Therefore, the MIC value of the *F. clypeata* water extract against *C. parapsilosis* was found to be 3.75 mg mL^−1^ (SI 2).

**Figure 3:**
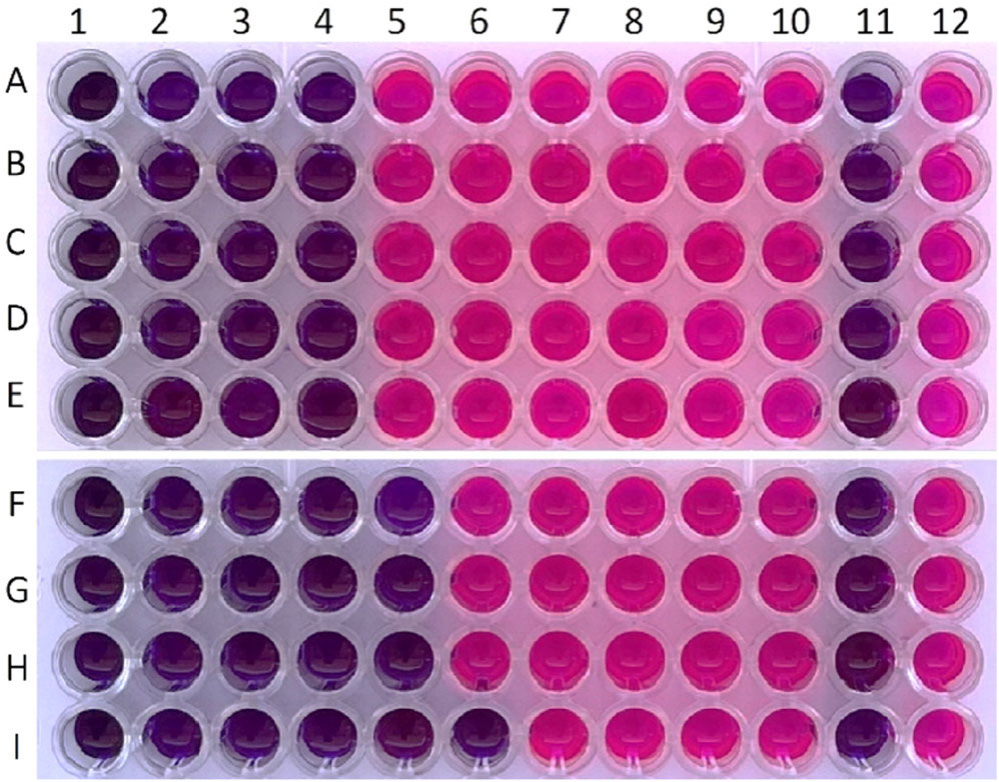
Microplate photo of *F. clypeata* water extracts antimicrobial activity assay against *S. aureus* A), *E. aerogenes* B), *E. coli* C)*, P. aeruginosa* D)*, K. pneumoniae* E)*, C. albicans* F)*, C. tropicalis* G)*, C. krusei* H)*, and C. parapsilosis* I). The water extract concentration in wells A4, B4, C4, D4, and E4 (15 mg/mL), F5, G5, and H5 (7.5 mg mL^−1^), and I6 (3.75 mg mL^−1^) without any color change were accepted as MIC values. The 11th and 12th wells are negative (including broth media) and positive control (including broth media and a microorganism inoculated in the same row), respectively.

The antimicrobial activity of *F. clypeata* water extract was repeated using the broth twofold microdilution method. *F. clypeata* water extract was added to the first wells on the microplate to obtain concentrations of 120 mg mL^−1^; then, twofold dilution was carried out for each strain. So, different microorganisms were treated with different concentrations of *F. clypeata* extracts in each row from the first to the tenth well. The MIC value was detected as *F. clypeata* water extract concentration with the least amount of the extract on the microplate on which the microdilution method is performed with no color change. The pink color reveals the growth of microorganisms in wells with resazurin included, and the blue represents growth inhibition. Consequently, same as the results of the agar dilution method, the MIC values of *F. clypeata* water extract against all bacterial strains used in the assay were found to be 15 mg mL^−1^ in the 4th wells without color change. The MIC values of *F. clypeata* water extract against *C. albicans, C. krusei*, and *C. tropicalis* were found to be 7.5 mg mL^−1^ in the 5th well, while it was 3.75 mg/mL against *C. parapsilosis* in the 6th well with no color change (**Figure** 2 and **3**). This experiment was replicated three times to increase the validity of the assay.

#### 4.2.2 Antimicrobial Activity of *F. clypeata* Methanol Extract

The antimicrobial assay results of *F. clypeata* methanol extract against bacterial strains are illustrated in SI 3. By observing the growth of microorganisms, MIC values of *F. clypeata* methanol extract were determined to be 60 (mg mL^−1^) against all bacterial species used (Figure 2). In addition, the antimicrobial assay results against yeast strains are demonstrated in SI 4. MIC values of *F. clypeata* methanol extract were determined to be 30 (mg mL^−1^) against *C. albicans, C. tropicalis*, and *C. krusei.* and 7.5 (mg mL^−1^) against *C. parapsilosis* (Figure 2).

**Figure 4:**
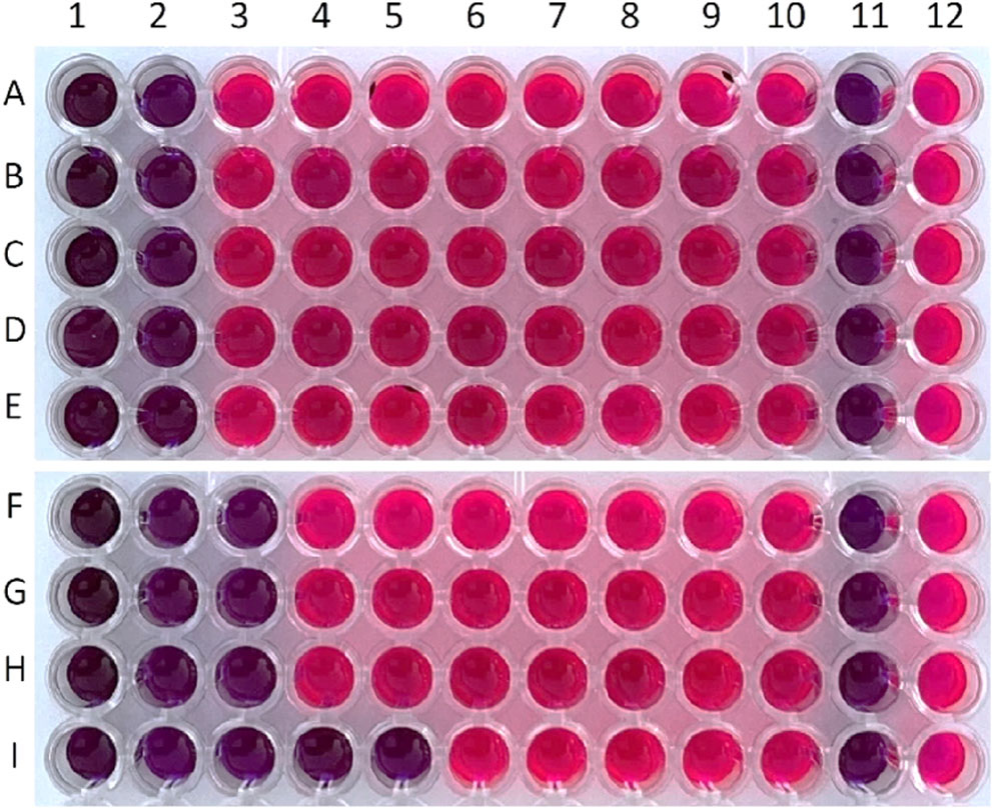
Microplate photo of *F. clypeata* methanol extracts antimicrobial activity assay against *S. aureus* A), *E. aerogenes* B), *E. coli* C)*, P. aeruginosa* D), *K. pneumoniae* E)*, C. albicans* F)*, C. tropicalis* G)*, C. krusei* H)*, and C. parapsilosis* I). The methanol extract concentration in wells A2, B2, C2, D2, and E2 (60 mg mL^‐1^), F3, G3, and H3 (30 mg mL^−1^), and I5 (7.5 mg mL^−1^) without any color change were accepted as MIC values. The 11th and 12th wells are negative (including broth media) and positive control (including broth media and a microorganism inoculated in the same row), respectively.

The antimicrobial activity of *F. clypeata* methanol extract was repeated using the broth microdilution method and demonstrated in **Figure** **4**. The results were the same as those of the agar dilution method. The MIC values of *F. clypeata* methanol extract against bacterial strains were determined to be 60 mg mL^−1^ in the second wells with no color change. The MIC values of *F. clypeata* methanol extract against *C. albicans, C. tropicalis*, and *C. krusei* were found to be 30 mg mL^−1^ in the third well, and it was found to be 7.5 mg mL^−1^ against *C. parapsilosis* in the fifth well (Figure 2 and 4). This experiment was replicated three times to verify the results.

### 4.3 The Cytotoxic Effects of *F. clypeata* in MM Cells

**Figure 5:**
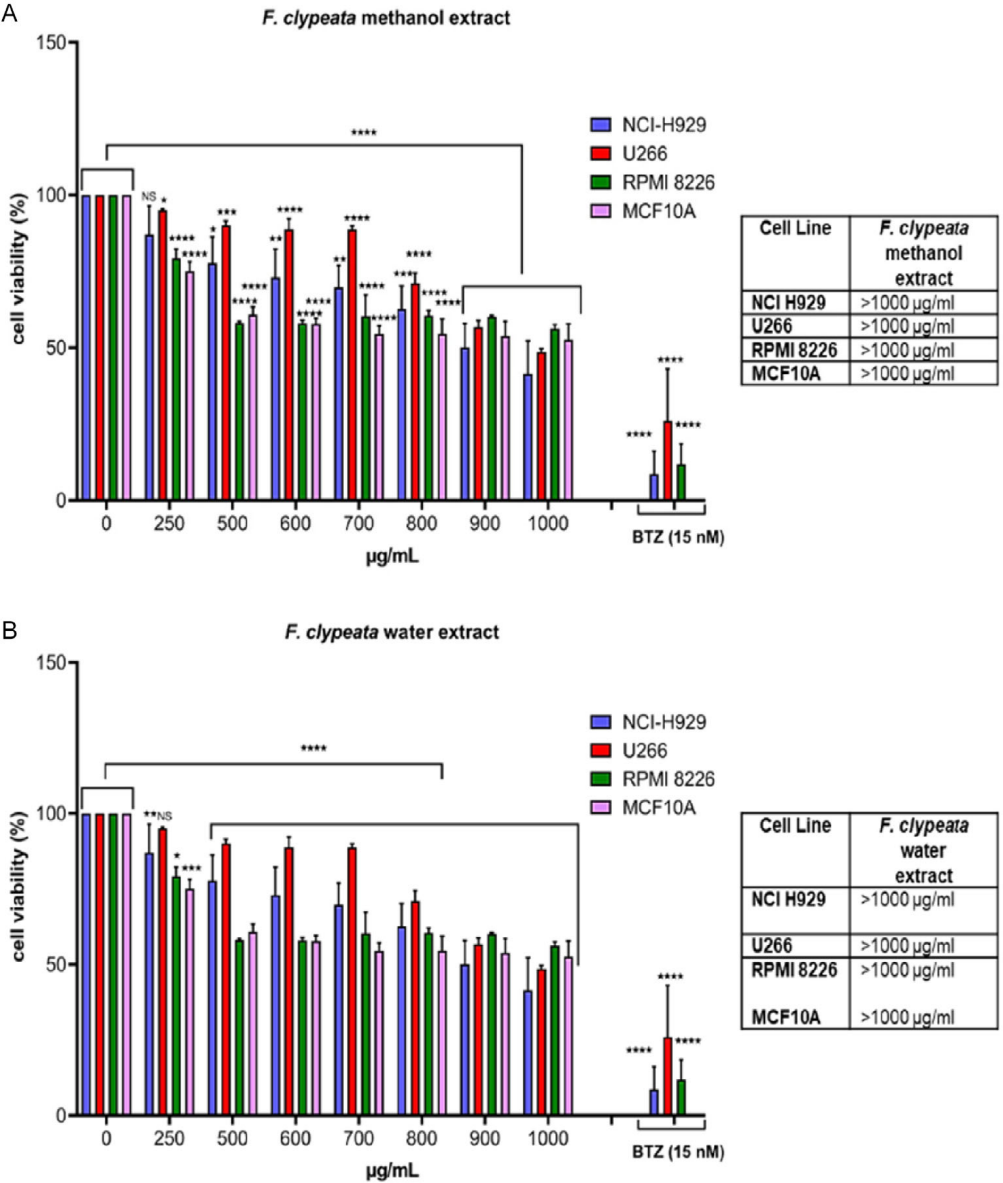
*F. clypeata* has cytotoxic effects on cells. MTT analysis in MM cell lines (NCI H929, U266, and RPMI 8226) and noncancerous MCF10A cells, treated with various concentrations of *F. clypeata* A) methanol extract and B) water extract for 48 hr. Error bars are mean ± SD. One‐way ANOVA followed by Dunnett's multiple comparison test was performed *p < 0.05, **p < 0.01, ***p < 0.001, ****p < 0.0001 (compared to the untreated group). Bortezomib (BTZ; 15 nM), a proteasome inhibitor, was used as a positive control.

To investigate the effect of *F. clypeata* on myeloma cell viability, we first dissolved *F. clypeata* in methanol and water with different concentrations. Next, we cultured MM and MCF10A cells with various concentrations of *F. clypeata* methanol or *F. clypeata* water extracts for 48 h. We also treated MM cell lines with the proteasome inhibitor Bortezomib (BTZ; 15 nM) for 48 h. BTZ is commonly used as a first‐line therapy in the clinical management of MM. Then, we assessed the cell viability using the MTT method. According to the MTT results, both methanol and water extracts of *F. clypeata* decreased cell viability in a dose‐dependent manner in all cell lines (**Figure** **5**A,B). Of note, both extracts of *F. clypeata* exhibited similar cytotoxic effects at a concentration of 900 µg mL^−1^ across all tested cell lines, including MM and noncancerous MCF10A cells. Regardless of the solvent used for extraction, *F. clypeata* markedly reduced cell viability in both MM and MCF10A cells. However, the IC_50_ values for *F. clypeata* were found to be greater than 1000 µg mL^−1^. Overall, it is emphasized that although similar cytotoxicity was observed, the dose of this extract should be carefully considered, as it exerts cytotoxic effects on both cancerous and noncancerous cells. Furthermore, additional studies using a broader panel of normal cell types and in vivo models are required to comprehensively assess the selectivity and safety of *F. clypeata*.

### 4.4 Results of in silico‐Molecular Docking Analysis

Most of the natural compounds from *F. clypeata* were energetically favored for the *S. aureus* MurB active site. Among them, cyanidin 3‐rutinoside chloride, cyanidin‐3‐O‐glycoside and rosmarinic acid were identified as prominent MurB inhibitory compounds with low binding free energy values of −9.4, 7.9, and 7.3 kcal mol^‐1^, respectively (**Table** **3**).

**Table 3:** Molecular docking outputs and interactive residue profiles of *F. clypeata* natural compounds with *S. aureus* MurB enzyme.

Compound ID	Binding energy 1HSK [kcal mol^−1^]	Interacting residues	*H bonds*
Chlorogenic acid	−7.2	Chain A: ALA154 TYR155 GLY156 TYR187 AGR225 LYS228 GLN229 SER238 PHE240 GLN241 ARG242 PRO243 PHE247 ALA248 GLY249 HIS271 PHE274	4
Cyanidin‐3‐O‐glucoside	−7.9	Chain A: GLY153 ALA154 TYR155 TYR187 ARG188 ARG225 GLN229 SER238 PHE240 GLN241 ARG242 PHE247 ALA248 GLY249 LYS250 HIS271 GLY273 GLU308	5
Ferulic acid	−6.2	Chain A: LEU17 VAL25 ALA37 LYS39 MET58 VAL67 ILE80 THE83 GLU83 TYR84 MET85 ASN86 GLY88 SER89 LEU137 ALA147	4
Fumaric acid	−4.3	Chain A: VAL25 ALA37 ILE38 LYS39 GLU54 MET58 VAL67 ILE80 THR82 GLU83 LEU137 ALA147 ASP148 PHE149	4
Cyanidin 3‐rutinoside chloride	−9.4	Chain A: ASN83 MET150 GLY153 ALA154 TYR155 TYR187 ARG188 ARG225 GLN229 GLY237 SER238 PHE240 GLN241 ARG242 PHE247 ALA248 GLY249 LYS250 HIS271 PHE274 GLU308	6
p‐Coumaric acid	−5.4	Chain A: ASN83 MET150 TYR187 ARG188 GLY237 SER238 VAL239 PHE240 GLN241 ARG242 GLU308	
Quinic acid	−5.6	Chain A: LEU17 VAL25 ALA37 ILE38 LYS39 GLU54 MET58 VAL67 ILE80 THR82 GLU83 TYR84 MET85 LEU137 ALA147 ASP148 PHE149	4
Rosmarinic acid	−7.3	Chain A: ASN83 MET150 TYR187 AGR188 GLY237 SER238 PHE240 GLN241 ARG242 PRO243 HIS246 PHE247 ALA248 GLY249 LYS250 HIS271 GLU308	7
Vanilic acid	−5.0	Chain A: ASN83 TYR187 ARG188 GLY237 SER238 PHE240 GLN241 ARG242 ALA248 GLU308	

**Figure 6:**
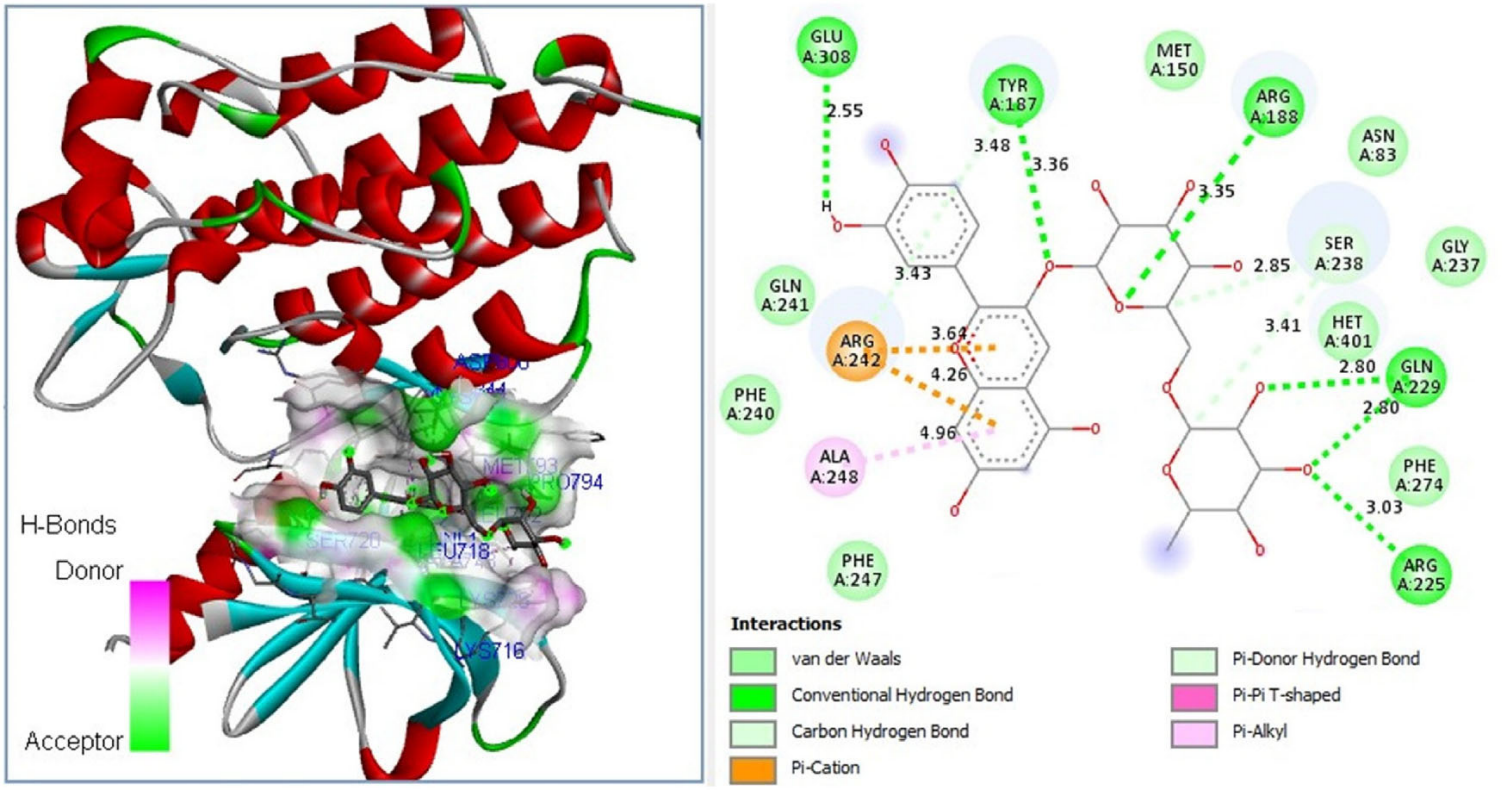
Molecular docking pose of cyanidin 3‐rutinoside chloride‐MurB (1HSK) and 2D depiction of their biochemical interaction potential.

Molecular docking studies revealed that the natural compound cyanidin 3‐rutinoside chloride exhibited a high binding affinity (Δ*G* = −9.4 kcal mol^−1^) to the catalytic site of the *S. aureus* MurB protein and showed the potential to introduce 6 hydrogen (H) bonds with 1HSK active site residues (**Figure** **6**).

Experimental findings also pointed out that cyanidin 3‐rutinoside chloride extracts were more active on *C. parapsilosis* fungi as compared to *C. albicans*. Therefore, comparative molecular docking analyses were performed on the DHFR enzymes of these two fungal pathogens. Related compounds were targeted to both *C. parapsilosis* and *C. albicans* DHFR enzymes through molecular docking studies to explain such specific affinity. Among the tested compounds, cyanidin 3‐rutinoside chloride revealed the highest binding affinity to the catalytic core of both *C. parapsilosis* and *C. albicans* DHFR targets with the lowest binding free energy of −9.3 and −8.7 kcal mol^−1^, respectively. Such similar selectivity on *C. parapsilosis* DHFR enzyme in the presence of lower binding free energy values was also identified for cyanidin‐3‐O‐glucoside, ferulic acid and rosmarinic acid compounds (**Table** **4**).

**Table 4:** Molecular docking outputs and interactive residue profiles of *F. clypeata* natural compounds with *C. parapsilosis* and *C. albicans* DHRF enzymes.

Compound ID	Binding energy C*. parapsilosis* model [Kcal mol^−1^]	Interacting residues	H bond	Binding energy 4HOE [Kcal mol^−1^]	Interacting residues	H bond
*Chlorogenic acid*	−7.9	ILE9 VAL10 ALA11 GLY23 SER24 LEU25 GLU32 MET33 PHE36 THR58 SER61 ILE62 PRO63 PHE66 LEU69 VAL113 ILE114 GLY115 TYR120 THR135	4	−7.9	ILE9 VAL10 ALA11 GLY23 LYS24 MET25 GLU32 PHE36 THR58 SER61 ILE62 PRO63 LEU69 ILE111 ILE112 GLY113 TYR118	3	
*Cyanidin‐3‐O‐glucoside*	−8.6	ILE9 VAL10 ALA11 GLY23 SER24 LEU25 TRP27 GLU32 MET33 PHE36 LYS37 THR58 SER61 ILE62 PRO63 PHE66 LEU69 PRO70 ARG72 VAL113 ILE114 GLY115 TYR120	3	−8.0	VAL10 ALA11 GLY23 LYS24 MET25 TRP27 LEU29 GLU32 ILE33 PHE36 LYS37 LYS57 THR58 SER61 ILE62 PRO63 PHE66 LEU69	3	
*Ferulic acid*	−6.6	ILE9 VAL10 ALA11 LEU25 GLU32 MET33 PHE36 THR58 SER61 ILE62 ILE114 TYR120	2	−6.3	ILE9 VAL10 ALA11 MET25 GLU32 PHE36 THR58 ILE62 LEU69 ILE112 TYR118 THR133	1	
*Fumaric acid*	−4.3	ILE9 VAL10 ALA11 LEU25 TRP27 GLU32 MET33 PHE36 ILE114 TYR120	1	−4.3	LYS31 GLU32 ARG34 TYR35 ASP38 THR171 VAL172 LEU173 GLU174 ILE177 TYR186	N/A	
*Cyanidin 3‐rutinoside chloride*	−9.3	ILE9 VAL10 ALA11 GLY23 SER24 LEU25 TRP27 ARG28 LEU29 ARG30 GLU32 MET33 PHE36 THR58 SER61 ILE62 PRO63 PHE66 LEU69 PRO70 ARG72 VAL113 ILE114 GLY115 TYR120	4	−8.7	ILE9 VAL10 ALA11 GLY23 LYS24 MET25 PRO26 TRP27 ARG28 LEU29 GLU32 ILE33 PHE36 LYS37 THR58 SER61 ILE62 PRO63 PHE66 LEU69 PRO70 ARG72 ILE112 TYR118	4	
*p‐Coumaric acid*	−6.4	ILE9 VAL10 ALA11 LEU25 GLU32 MET33 PHE36 MET54 THR58 TRP59 SER61 ILE62 ILE114 TYR120	1	−6.5	ILE9 VAL10 ALA11 MET25 GLU32 PHE36 THR58 ILE62 LEU69 ILE112 TYR118 THR133	1	
*Quinic acid*	−5.5	ILE9 VAL10 ALA11 LEU25 TRP27 GLU32 MET33 PHE36 THR58 ILE62 ILE114 GLY115 TYR120 THR135	4	−5.6	LYS31 GLU32 ARG34 TYR35 ASP38 THR171 VAL172 LEU173 GLU174 ILE177 TYR186	3	
*Rosmarinic acid*	−8.7	ILE9 VAL10 ALA11 LEU25 GLU32 MET33 PHE36 LYS37 THR58 SER61 ILE62 PRO63 PHE66 LEU69 PRO70 ARG72 ILE114 GLY115 TYR120	5	−7.7	ILE9 VAL10 ALA11 MET25 GLU32 ILE33 PHE36 THR58 ILE62 PRO63 PHE66 LEU69 ILE111 ILE112 GLY113 TYR118 THR133	1	
*Vanilic acid*	−5.6	ILE9 ALA11 LEU25 TRP27 GLU32 MET33 PHE36 THR58 SER61 ILE62 ILE114 GLY115 TYR120	2	−6.3	ILE9 VAL10 ALA11 MET25 GLU32 ILE33 PHE36 ILE112 TYR118 THR133	2	
18G	−8.2	ILE9 LEU25 MET33 PHE36 LYS37 SER61 ILE62 PRO63 PHE66 LEU69 PRO70 ARG72 ILE114 GLY115 TYR120	1	−7.9	ILE9 VAL10 ALA11 MET25 GLU32 ILE33 PHE36 LYS37 THR58 SER61 ILE62 PRO63 PHE66 LEU69 PRO70 ARG72 ILE112 GLY113 TYR118 THR133	2	

**Figure 7:**
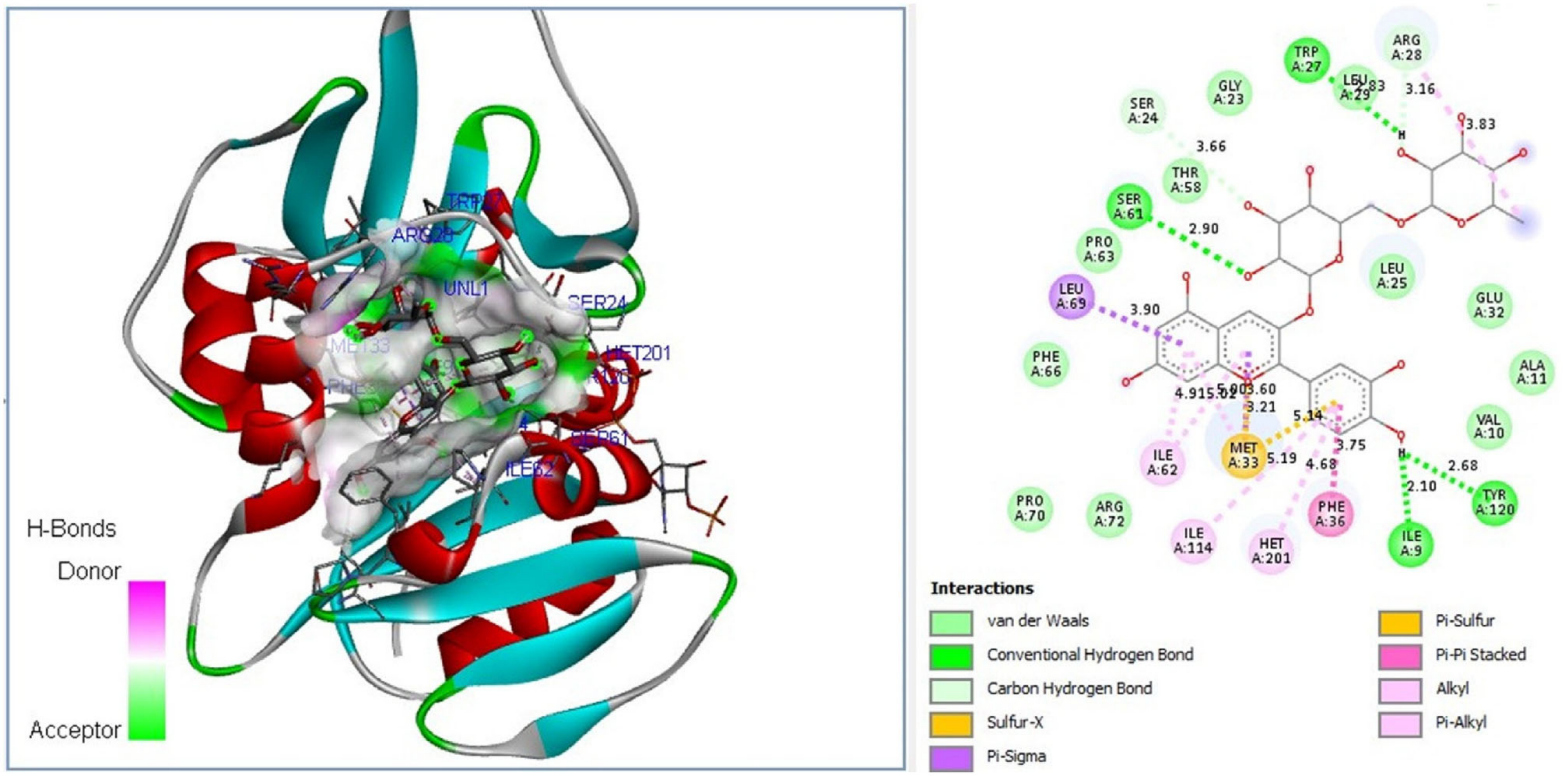
Molecular docking pose of cyanidin 3‐rutinoside chloride‐*C. parapsilosis* DHFR model and 2D depiction of their biochemical bond interactions.

**Figure 8:**
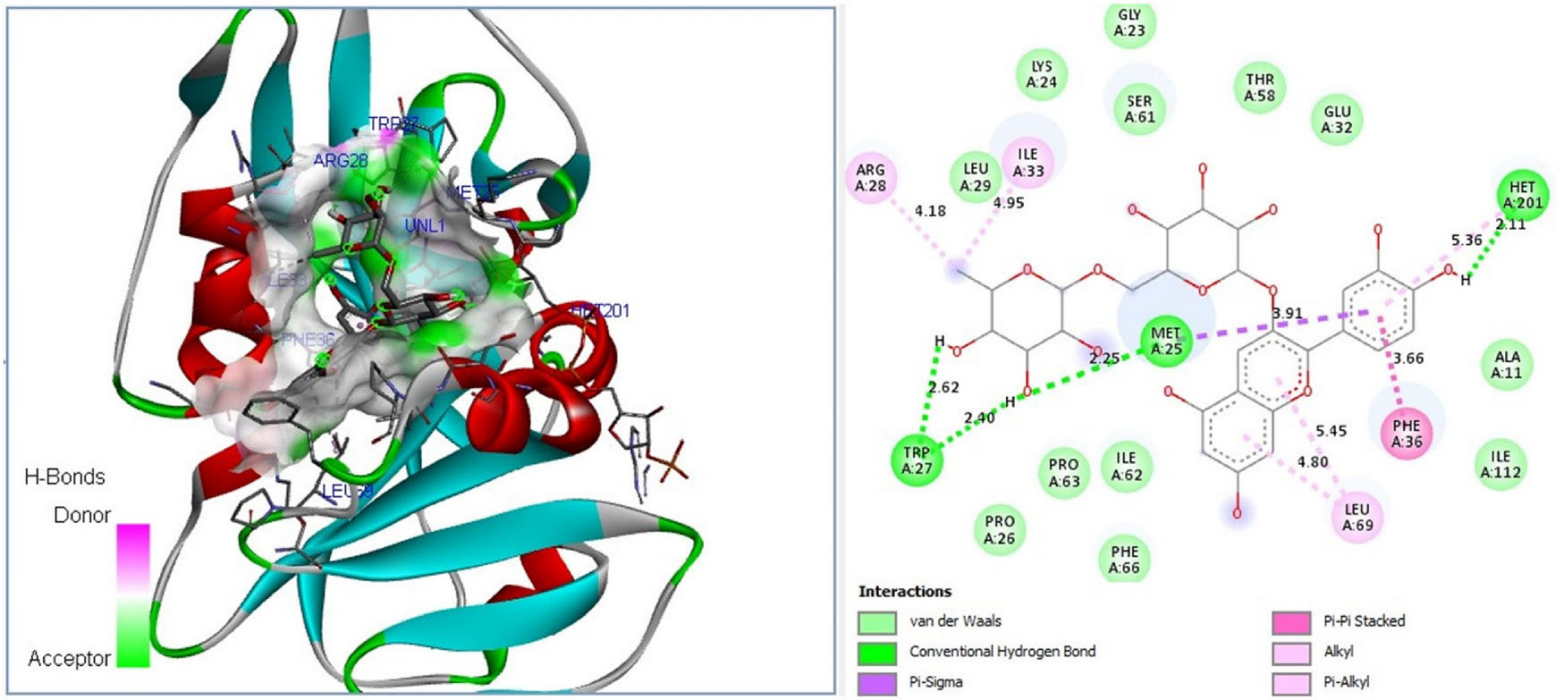
Molecular docking pose of cyanidin 3‐rutinoside chloride‐*C. albicans* DHFR model and 2D depiction of their biochemical bond interactions.

Experimental findings further revealed that natural metabolites of *F. clypeata* showed higher antifungal activity against *C. parapsilosis* than against *C. albicans*. Comparative molecular docking studies were thus performed on the DHFR enzymes of both fungi to determine whether this result was due to the binding affinity difference of *F. clypeata* metabolites. Molecular docking outputs on the antifungal potential of *F. clypeata* natural compounds revealed that cyanidin 3‐rutinoside chloride displayed the highest binding affinity to the catalytic core of both *C. parapsilosis* and *C. albicans* DHFR. 2D plot analysis results extracted from molecular docking data further confirmed that cyanidin 3‐rutinoside has the potential to build 6 strong hydrogen bonds with catalytic residues of *C. parapsilosis* DHFR enzyme (**Figure** **7**), whereas the total hydrogen bond building potential with catalytic residues of *C. albicans* DHFR enzyme is only 4 (**Figure** **8**).

**Figure 9:**
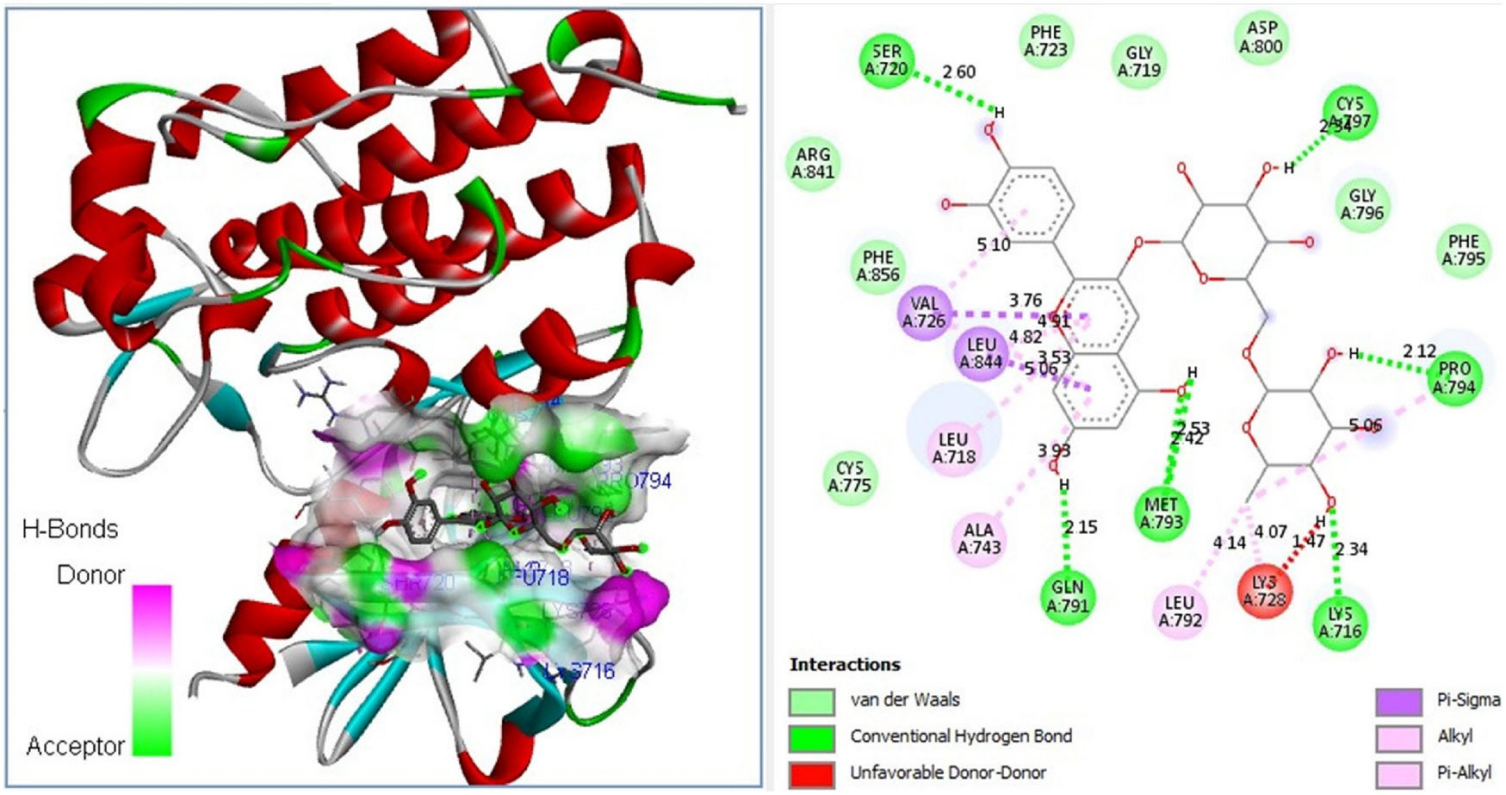
Proposed molecular docking pose of cyanidin 3‐rutinoside chloride‐human EGFR (PDB ID: 5UG9) and 2D depiction of their biochemical bond interactions.

The antitumorigenic activities of *F. clypeata* metabolites were also investigated based on their binding affinity to human EGFR protein (**Table** **5**). Among nine natural metabolites of *F. clypeata* extracts, the cyanidin 3‐rutinoside chloride compound from *F. clypeata* was again identified with the highest binding affinity to the catalytic core of 5UG9 protein. Furthermore, molecular docking analysis indicated that cyanidin 3‐rutinoside chloride could potentially form 7 H bonds with 5UG9 catalytic core residues (**Figure** **9**). The high binding affinity of cyanidin 3‐rutinoside chloride to the EGFR target further indicated its promising antifungal effect on MM cell lines. Furthermore, cyanidin‐3‐O‐glucoside and rosmarinic acid were also identified to have high binding affinity to human EGFR protein (Table 5).

**Table 5:** Molecular docking outputs and interactive residue profiles of *F. clypeata* natural compounds with human EGFR protein.

Compound ID	Binding energy 5UG9 [Kcal mol^−1^]	Interacting residues	H bond
*Chlorogenic acid*	−7,8	Chain A: LEU718 GLY719 SER720 GLY721 PHE723 VAL726 ALA743 CYS775 MET790 GLN791 LEU792 MET793 CYS797 ARG841 ASN842 LEU844 THR854 PHE856	4
*Cyanidin‐3‐O‐glucoside*	−8,8	Chain A: LEU718 GLY719 SER720 GLY721 PHE723 VAL726 ALA743 CYS775 MET790 GLN791 LEU792 MET793 PRO794 PHE795 GLY796 CYS797 ASP800 ARG841 ASN842 LEU844 THR854 PHE856	4
*Ferulic acid*	−6,5	Chain A: LEU718 VAL726 ALA743 LYS745 MET790 GLN791 LEU792 MET793 PRO794 PHE795 GLY796 CUS797 LEU844 THR854 ASP855 PHE856	4
*Fumaric acid*	−4,2	Chain A: LEU718 VAL726 ALA743 LYS745 MET790 GLN791 LEU792 MET793 PRO794 PHE795 GLY796 CUS797 LEU844 THR854 ASP855 PHE856	5
*Cyanidin 3‐rutinoside chloride*	−9,4	Chain A: LYS716 LEU718 GLY719 SER720 GLY721 PHE723 VAL726 LYS728 ALA743 CYS775 MET790 GLN791 LEU792 MET793 PRO794 PHE795 GLY796 CYS797 ASP800 ARG841 ASN842 LEU844 LEU844 THR854 PHE856	7
*p‐Coumaric acid*	−6,3	Chain A: LEU718 VAL726 ALA743 LYS745 MET790 GLN791 LEU792 MET793 PRO794 PHE795 GLY796 CUS797 LEU844 THR854 ASP855 PHE856	4
*Quinic acid*	−5,2	Chain A: LEU718 VAL726 ALA743 CYS775 MET790 GLN791 LEU792 MET793 PRO794 PHE795 GLY796 CYC797CLEU844 THR854 PHE856	2
*Rosmarinic acid*	−8,2	Chain A: LEU718 GLY719 SER720 PHE723 VAL726 ALA743 LYS745 CYS775 ARG776 MET790 GLN791 LEU792 MET793 PRO794 PHE795 GLY796 CYC797 ASP800 ARG841 LEU844 THR854 PHE856	3
*Vanilic acid*	−5,8	Chain A: LEU718 VAL726 ALA743 LYS745 CYC775 MET790 GLN791 LEU792 MET793 PRO794 GLY796 CYC797 LEU844 THR854 PHE856	1
*8AM*	−8,9	Chain A: LEU718 GLY719 SER720 PHE723 VAL726 ALA743 LYS745 MET790 GLN791 LEU792 MET793 PRO794 PHE795 GLY796 CYS797 ASP837 ARG841 ASN842 LEU844 THR854 PHE856	1

In numerous studies over the years, it has been reported that the bioactive properties of the plant are determined by the phenolics it contains.^[^ ^32^, ^33^, ^34^, ^35^ ^–^ ^36^ ^]^ Studies on the pharmacological effects of *F. clypeata* are pretty limited. Zengin et al. determined the contents of *F. clypeata* extracts by HPLC‐ESI‐QTOF‐MS analysis. Unlike this study, their analysis also focused on glucosinolates; however, the percentages of the compounds detected in the extracts were not reported.^[^ ^8^ ^]^ As a result of LC‐HR/MS analysis, phenolic compounds defined in plant content affected antimicrobial and antimyeloma results in this study. By LC‐HR/MS analysis, nine phenolic compounds were discovered as a result of our extraction investigation with *F. clypeata*; fumaric acid (3.66 g kg^‐1^) was the most common in water extract, and ferulic acid (1.73 g kg^‐1^) was the most common in methanol extract. Even though more phenolic compounds flow into the water extract, the amount of chemicals in the methanol extract is smaller. This is due to the low number of carbons in the structures of these molecules and the presence of functional groups such as phenol and carboxylic acid, which can form extra hydrogen bonds, increasing solubility in water (SI 5). Chlorogenic acid and quinic acid were found in both extracts in trace amounts.

As a result of antimicrobial studies, the water extract had a more potent inhibition against microorganisms than the methanol extract. The MIC value of the water extract against *S. aureus, E. aerogenes, E. coli, K. pneumoniae*, and *P. aeruginosa* is 15 mg mL^−1^, against *C. albicans, C. tropicalis*, and *C. krusei* is 7.5 mg/mL, and against *C. parapsilosis* is 3.75 mg mL^−1^. However, interestingly, the inhibitory efficiency of the methanol extract decreased very regularly. The MIC values of *F. clypeata* methanol extract against bacterial microorganisms are 60 mg/mL; against *C. tropicalis, C. albicans*, and *C. krusei* is 30 mg/mL; and against *C. parapsilosis* is 7.5 mg mL^−1^. The difference in inhibition values between methanol and water extracts can be attributed to the differences in phenolic compounds they contain and the amounts of these compounds.

Among all the identified natural compounds from *F. clypeata*, cyanidin 3‐rutinoside chloride was proposed to have the highest antifungal and promising anticancer activities on MM cell lines. Molecular docking data provided significant evidence that among all the analyzed natural compounds, cyanidin 3‐rutinoside chloride could be pronounced as one of the new natural inhibitors of human EGFR in the near future. Therefore, we anticipate that it would be valuable to investigate the theoretical findings regarding the high binding affinity of the natural compound cyanidin 3‐rutinoside chloride against *S. aures* MurB, *Candida* DHFR, and the antitumorigenic target EGFR with further experimental studies.

As a result of LC‐HR/MS analysis, it was determined that the quantitative amount of fumaric acid, cyanidin 3‐rutinoside chloride, vanillic acid, p‐coumaric acid, and ferulic acid is higher in the water extract than in the methanol extract. Fumaric acid is almost 15 times more present in water extract (3.6649 g kg^‐1^) compared to methanol extract (0.2454 g kg^‐1^). Although there are not enough studies related to the antimicrobial activity of fumaric acid in the literature, its effectiveness has been observed in studies combined with chitosan or silver chloride nanocomposites.^[^ ^37^, ^38^ ^]^ Fumaric acid gains antimicrobial properties due to its double‐bond structure and two carboxylic groups.^[^ ^39^ ^]^ In this study, cyanidin 3‐rutinoside chloride in the water extract (0.5546 g kg^‐1^) is more than two times the amount in the methanol extract (0.2412 g kg^‐1^). Although the mechanism of action has yet to be explained, the antimicrobial activity of some plants containing cyanidin 3‐rutinoside chloride has been reported previously.^[^ ^40^, ^41^ ^–^ ^42^ ^]^ On the other hand, in silico molecular docking analyses further pointed out that the presence of cyanidin 3‐rutinoside chloride in higher quantities might be the main reason why *F. clypeata* water extracts exhibited better antibacterial, antifungal, and anticancer activities compared to methanol extracts. In addition, the availability of cyanidin 3‐rutinoside chloride in higher quantities further explains the better antifungal activity of *F. clypeata* water extracts. Furthermore, consistent with the molecular docking data, the higher antifungal activity of *F. clypeata* water extracts on *C. parapsilosis* rather than *C. albicans* is most likely due to the higher binding affinity of cyanidin 3‐rutinoside chloride to the *C. parapsilosis* DHFR enzyme.

The vanillic acid in the water extract (0.6885 g kg^‐1^) was almost twice that in the methanol extract (0.3743 g kg^‐1^). In another study with vanillic acid, it was reported that the antimicrobial effect of phenolic acid derivatives increases as the length of the alkyl chain increases.^[^ ^9^ ^]^ Vanillic acid has a bactericidal effect depending on the PH value of the medium created.^[^ ^43^ ^]^ According to Santos Oliveira et al., vanillic acid derivatives show activity by modifying cell wall functions and plasma membranes in fungal species.^[^ ^44^ ^]^ P‐coumaric acid has a dual antimicrobial mechanism. P‐coumaric acid significantly increases the outer and plasma membrane permeability, resulting in loss of membrane function.^[^ ^45^ ^]^ At the same time, it inserts into the groove in the DNA double helix by binding to the phosphate anion in the DNA double helix. As a result, it inhibits cellular functions by affecting replication and transcription. All these changes made by p‐coumaric acid result in cell death.^[^ ^45^ ^]^ P‐coumaric acid therapy significantly damages cell integrity by reducing intracellular ATP, cell membrane hyperpolarization, cell morphology malformation, and whole‐cell protein disruption.^[^ ^46^ ^]^ Ferulic acid causes a decrease in intracellular ATP concentration. At the same time, it causes a decrease in intracellular pH and hyperpolarization of the cell membrane. Ferulic acid causes changes in membrane integrity, dysfunction by damaging the cell membrane, and changes in cellular morphology.^[^ ^47^, ^48^ ^]^

In this study, close amounts of cyanidin‐3‐O‐glucoside (0.3390 and 0.5435 g kg^‐1^, respectively) and rosmarinic acid (0.1057 and 0.1279 g kg^‐1^, respectively) were found when water and methanol extracts were compared. In the literature, although there are various studies on the mechanism of action and functional structure of cyanidin‐3‐O‐glucoside, there is no precise study on the presence of its direct antimicrobial effect.^[^ ^49^, ^50^ ^–^ ^51^ ^]^ However, many studies proving the antimicrobial effect of rosmarinic acid have been found in the literature.^[^ ^52^, ^53^, ^54^, ^55^ ^–^ ^56^ ^]^ Rosmarinic acid prevents microbial cells from attaching and reduces biofilm formation.^[^ ^53^, ^57^ ^]^ Rosmarinic acid exerts antifungal activity by reducing mitochondrial activity, causing changes in membrane integrity, and inhibiting protease production.^[^ ^52^ ^]^ In this work, albeit in minimal amounts, quinic acid, present in both water and methanol extracts (0.0022 and 0.0560 g kg^‐1^, respectively), showed antimicrobial properties. Quinic acid directs the functions of ribosomes and the synthesis of aminoacyl‐tRNAs. It also alters the glycerophospholipids and fatty acids levels and alters membrane fluidity by disrupting the oxidative phosphorylation pathway.^[^ ^58^, ^59^ ^]^ In this study, although the chlorogenic acid in the methanol extract is found in minimal amounts, studies have proven it has significant antimicrobial activity.^[^ ^60^, ^61^ ^]^ Chlorogenic acid is a metabolite obtained from herbal products and has proven antimicrobial activity. Chlorogenic acid causes a decrease in intracellular ATP concentrations and lowers the ambient pH, resulting in cell membrane hyperpolarization and eliminating cell membrane permeability.^[^ ^60^, ^61^ ^]^

Regardless of the extraction method, *F. clypeata* displayed similar cytotoxicity in all MM cell lines and the noncancerous mammary gland MCF10A cell line. Results obtained from previous studies indicated that herbal extracts could display anticancer activity towards MM. In parallel with our results, it was shown that bavachin, a phytoestrogen purified from natural herbal plants, decreased MM cell viability but not normal cells.^[^ ^62^ ^]^ Furthermore, apigenin, one of the most abundant dietary flavonoids, had a cytotoxic effect on MM cell lines by suppressing COX‐2 and iNOS expression in STAT1‐transfected HEK293 cells.^[^ ^63^ ^]^ The study of Yu et al. indicates that resveratrol, curcumin, daidzin, gambogic acid, baicalein, cinobufagin, ginsenoside, berberine, formononetin, bufalin, icariin, polysaccharides extracts from Hedyotis difus, and scutellarein demonstrated anti‐MM effects via the regulation of apoptosis, proliferation, osteogenic differentiation, autophagy, drug resistance, and cell cycle.^[^ ^64^ ^]^ The MTT results of the present study show the cytotoxic effect of *F. clypeata* in either MM cells or noncancerous cells, indicating its careful use as a pharmacotherapeutic compound.

## 5 Conclusion

In this study, in vitro *a*ntimicrobial and antimyeloma properties of *F. clypeata* extracts were investigated for the first time, and in silico molecular docking analyses were performed on the identified secondary metabolites. As a result of the antimicrobial assay, it was observed that water and methanol extracts had significant inhibition values. However, the inhibitory effect of the methanol extract on microorganisms decreased very regularly compared to the water extract. These data indicated the potential application of *F. clypeata* water and methanol extracts as natural antimicrobial agents for treating infections caused by bacteria and *Candida* strains. However, using the antimyeloma property of *F. clypeata* water and methanol extracts against MM requires more attention due to its possible cytotoxic effects on normal cells. This study opens up exciting possibilities for its use in medicine, pharmacy, and the food industry, warranting further in vivo studies to fully explore its promising applications.

## Conflict of Interest

The authors declare no conflicts of interest.

## Author Contributions

**Tuba Unver**: conceptualization (lead); data curation (equal); formal analysis (equal); investigation (equal); methodology (equal); project administration (lead); supervision (lead); validation (equal); visualization (equal); writing—original draft (lead); writing—review and editing (lead). **Ugur Uzuner**: data curation (equal); formal analysis (equal); investigation (equal); methodology (equal); software (lead); validation (equal); visualization (equal); writing—original draft (equal); writing—review and editing (equal). **Dilara Akcora‐Yildiz**: data curation (equal); formal analysis (equal); investigation (equal); methodology (equal); software (equal); validation (equal); visualization (equal); writing—original draft (equal); writing—review and editing (equal). **Ismet Gurhan**: data curation (equal); formal analysis (equal); investigation (equal); methodology (equal); validation (supporting). **Caglar Arkan**: data curation (supporting); formal analysis (supporting); investigation (equal); methodology (supporting). **Zeynep Ozdemir**: formal analysis (supporting); investigation (supporting); methodology (supporting); validation (supporting).

## Supporting information

Supplementary Material

## Data Availability

The data that support the findings of this study are available from the corresponding author upon reasonable request.
