# Supplementary material for: Multidirectional in silico and in vitro Research for the Pharmaceutical Potential of Fibigia Clypeata (L.) Medik: Phytochemical, Antimicrobial, and Antimyeloma Properties
Source: ChemistryOpen. 2025 Sep 4;14(12):e202500036. doi: 10.1002/open.202500036 (PMC12680575; doi:10.1002/open.202500036)
Supplement: Supplementary file 1 — Supplementary Material [file OPEN-14-e202500036-s001.zip › OPEN-202500036-sup-0001-suppdata-S1/Supporting Information 1.pdf]

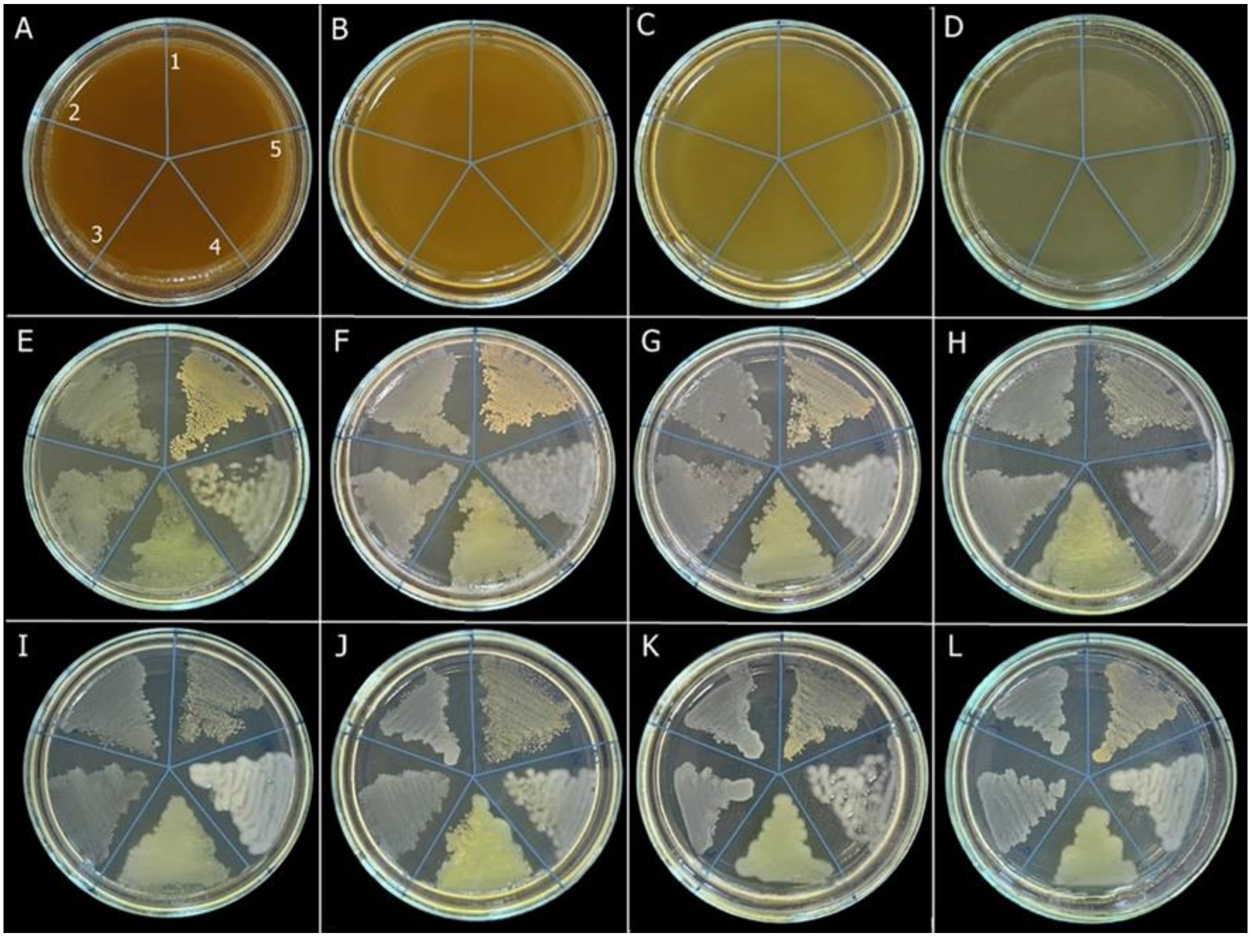

Supporting Information 1. Results of the antibacterial activity assay of *F. clypeata* water extract with different concentrations against *S. aureus* (1), *E. aerogenes* (2), *E. coli* (3), *P. aeruginosa* (4), and *K. pneumoniae* (5). (A) 120, (B) 60, (C) 30, (D) 15, (E) 7.5 (F) 3.75, (G) 1.87, (H) 0.94, (I) 0.47, (J) 0.23, (K) 0.12 mg/mL *F. clypeata* water extract, (L) Control

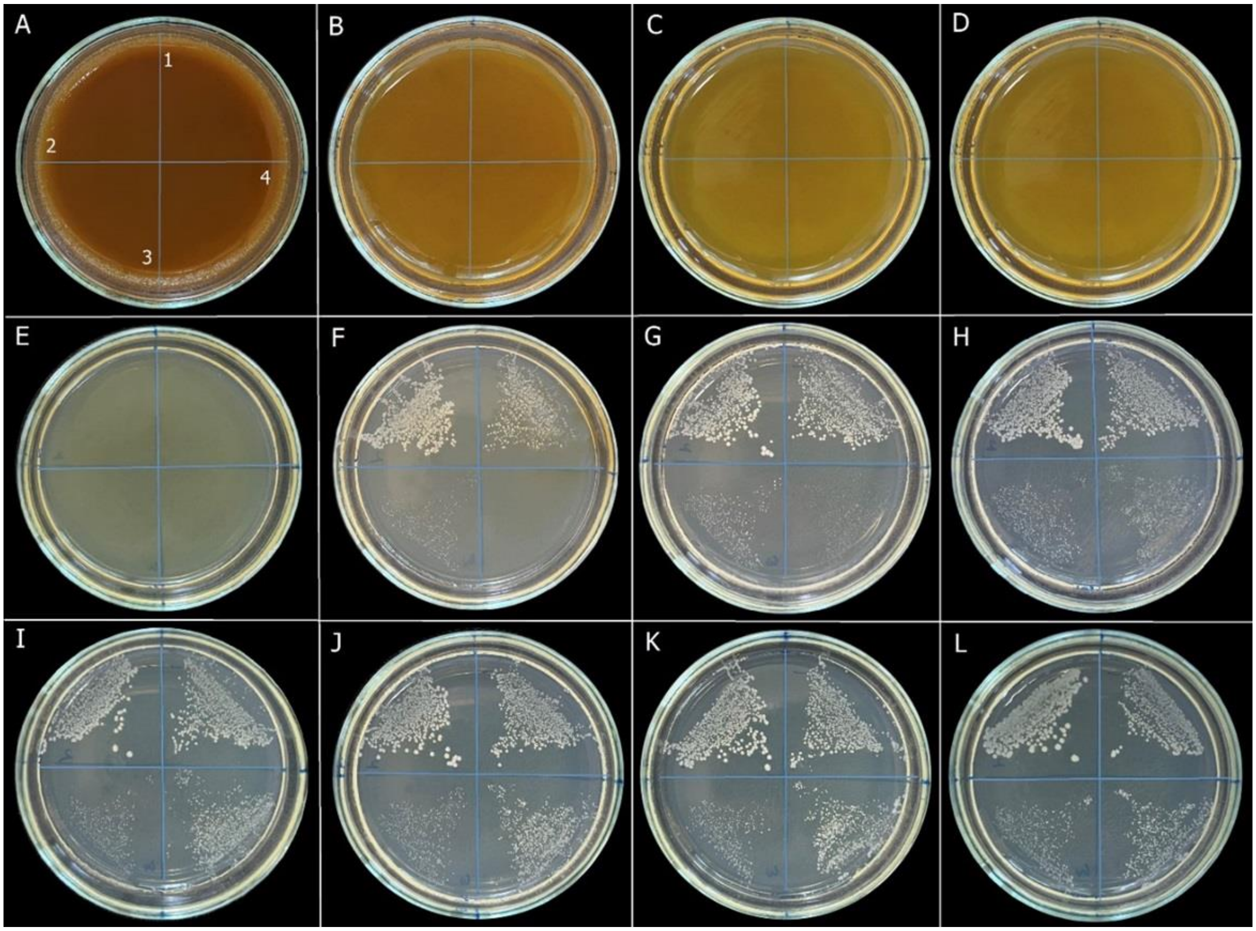

Supporting Information 2. Results of the antifungal activity assay of *F. clypeata* water extract with different concentrations against *C. albicans* (1), *C. tropicalis* (2), *C. krusei* (3), *and C. parapsilosis* (4). (A) 120, (B) 60, (C) 30, (D) 15, (E) 7.5 (F) 3.75, (G) 1.87, (H) 0.94, (I) 0.47, (J) 0.23, (K) 0.12 mg/mL *F. clypeata* water extract, (L) Control

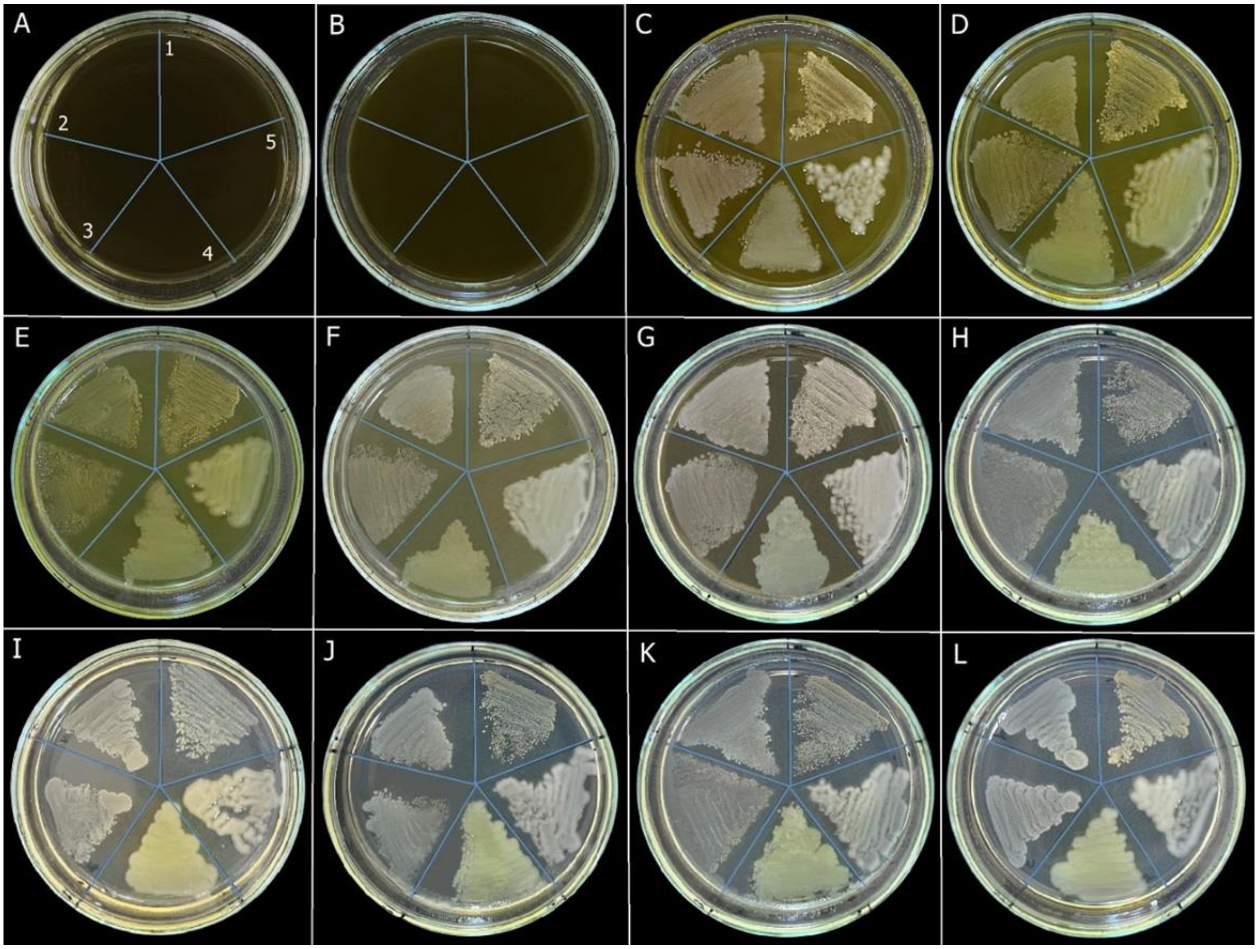

Supporting Information 3. Results of the antibacterial activity assay of *F. clypeata* methanol extract with different concentrations against *S. aureus* (1), *E. aerogenes* (2), *E. coli* (3), *P. aeruginosa* (4), and *K. pneumoniae* (5). (A) 120, (B) 60, (C) 30, (D) 15, (E) 7.5 (F) 3.75, (G) 1.87, (H) 0.94, (I) 0.47, (J) 0.23, (K) 0.12 mg/mL *F. clypeata* methanol extract, (L) Control

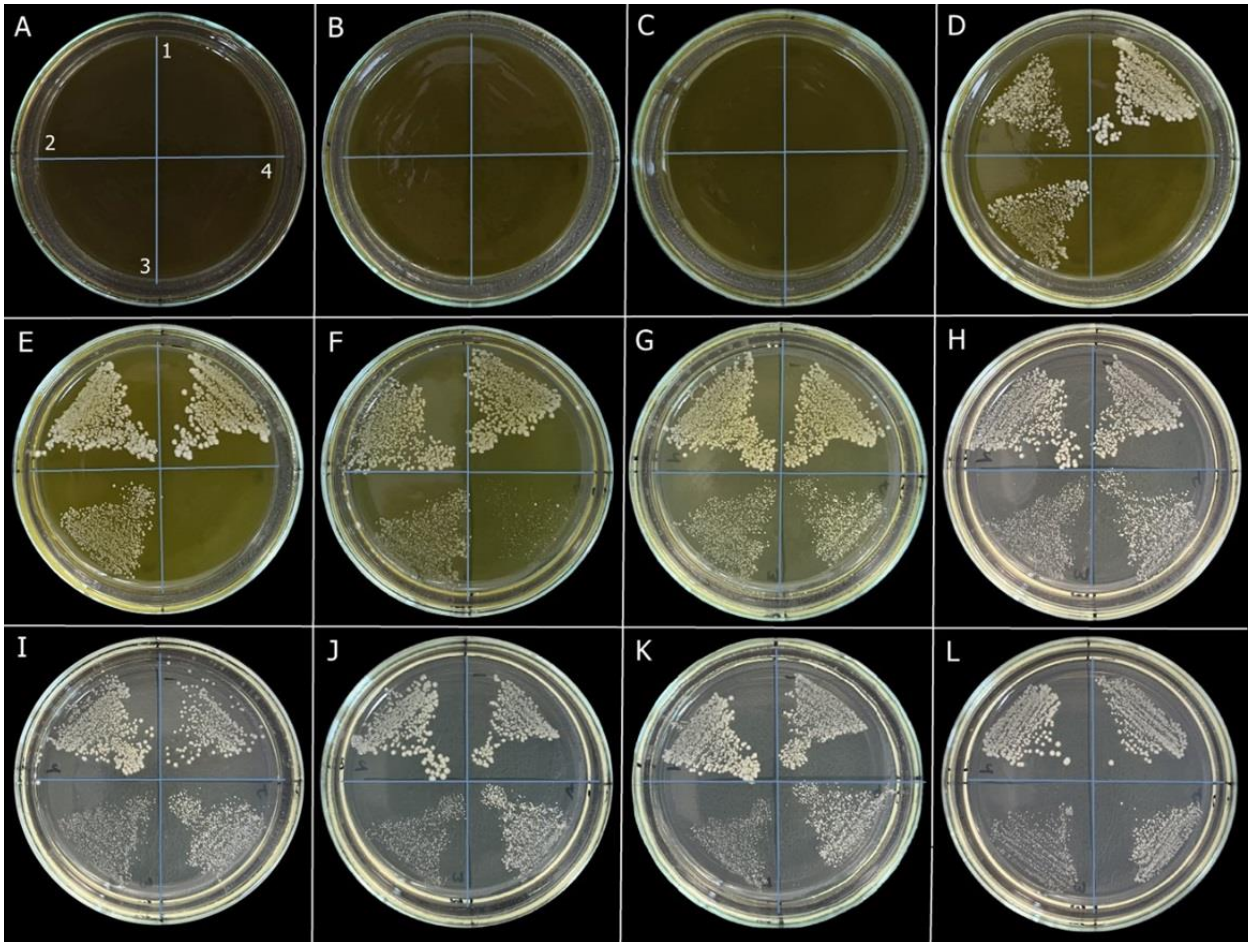

Supporting Information 4. Results of the antifungal activity assay of *F. clypeata* methanol extract with different concentrations against *C. albicans* (1), *C. tropicalis* (2), *C. krusei* (3), *and C. parapsilosis* (4). (A) 120, (B) 60, (C) 30, (D) 15, (E) 7.5 (F) 3.75, (G) 1.87, (H) 0.94, (I) 0.47, (J) 0.23, (K) 0.12 mg/mL *F. clypeata* methanol extract, (L) Control

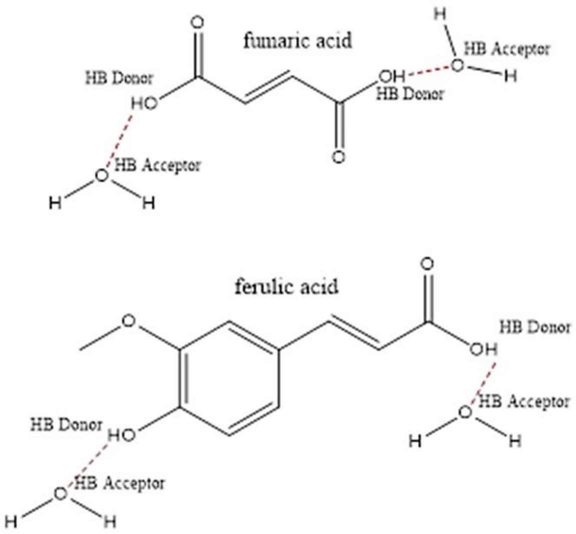

Supporting Information 5. Hydrogen bonding donors (HBD) of phenolic molecules in water extract.
