# Supplementary material for: Multidirectional in silico and in vitro Research for the Pharmaceutical Potential of Fibigia Clypeata (L.) Medik: Phytochemical, Antimicrobial, and Antimyeloma Properties
Source: ChemistryOpen. 2025 Sep 4;14(12):e202500036. doi: 10.1002/open.202500036 (PMC12680575; doi:10.1002/open.202500036)
Supplement: Supplementary file 1 — Supplementary Material [file OPEN-14-e202500036-s001.zip › OPEN-202500036-sup-0001-suppdata-S1/Supporting Information 2.pdf]

Scan Segments

Cpd Name	ISTD?	Prec Ion	MS1 Res	Prod Ion	MS2 Res	Dwell	Frag (V)	CE (V)	Cell Acc (V)	Polarity
Hesperidin	No	609	Unit/Enh (6490)	300.9	Unit/Enh (6490)	10	90	22	4	Negative
Keracyanin Chloride	No	592.8	Unit/Enh (6490)	284.7	Unit/Enh (6490)	10	90	28	4	Negative
Naringin	No	579	Unit/Enh (6490)	458.6	Unit/Enh (6490)	10	170	26	4	Negative
Naringin	No	579	Unit/Enh (6490)	270.8	Unit/Enh (6490)	10	170	34	4	Negative
Peonidin-3-o-glucoside	No	460.9	Unit/Enh (6490)	298.8	Unit/Enh (6490)	10	130	16	4	Negative
Epigallocatechin Gallate	No	456.8	Unit/Enh (6490)	304.9	Unit/Enh (6490)	10	110	16	4	Negative
Epigallocatechin Gallate	No	456.8	Unit/Enh (6490)	168.8	Unit/Enh (6490)	10	110	14	4	Negative
Cyanidin-3-o-glucoside	No	447.1	Unit/Enh (6490)	284.8	Unit/Enh (6490)	10	130	18	4	Negative
Cyanidin-3-o-glucoside	No	447.1	Unit/Enh (6490)	283.8	Unit/Enh (6490)	10	130	26	4	Negative
Vitexin	No	430.9	Unit/Enh (6490)	340.6	Unit/Enh (6490)	10	140	22	4	Negative
Vitexin	No	430.9	Unit/Enh (6490)	310.9	Unit/Enh (6490)	10	140	22	4	Negative
Curcumin	No	366.9	Unit/Enh (6490)	216.9	Unit/Enh (6490)	10	90	8	4	Negative
Curcumin	No	366.9	Unit/Enh (6490)	148.9	Unit/Enh (6490)	10	90	14	4	Negative
Rosmarinic Acid	No	358.8	Unit/Enh (6490)	196.6	Unit/Enh (6490)	10	90	14	4	Negative
Rosmarinic Acid	No	358.8	Unit/Enh (6490)	160.8	Unit/Enh (6490)	10	90	14	4	Negative
Chlorogenic Acid	No	352.9	Unit/Enh (6490)	190.9	Unit/Enh (6490)	10	80	14	4	Negative
Myricetin	No	316.9	Unit/Enh (6490)	178.8	Unit/Enh (6490)	10	90	18	4	Negative
Myricetin	No	316.9	Unit/Enh (6490)	150.9	Unit/Enh (6490)	10	90	24	4	Negative
Isorhamnetin	No	314.9	Unit/Enh (6490)	299.8	Unit/Enh (6490)	10	90	20	4	Negative
Isorhamnetin	No	314.9	Unit/Enh (6490)	150.6	Unit/Enh (6490)	10	90	30	4	Negative
Taxifolin	No	302.9	Unit/Enh (6490)	284.9	Unit/Enh (6490)	10	90	8	4	Negative
Taxifolin	No	302.9	Unit/Enh (6490)	124.7	Unit/Enh (6490)	10	90	22	4	Negative
Quercetin	No	300.9	Unit/Enh (6490)	178.7	Unit/Enh (6490)	10	110	16	4	Negative
Quercetin	No	300.9	Unit/Enh (6490)	150.7	Unit/Enh (6490)	10	110	20	4	Negative
Ellagic Acid	No	300.8	Unit/Enh (6490)	283.4	Unit/Enh (6490)	10	160	34	4	Negative
Ellagic Acid	No	300.8	Unit/Enh (6490)	228.7	Unit/Enh (6490)	10	160	28	4	Negative
Catechin	No	289.1	Unit/Enh (6490)	244.9	Unit/Enh (6490)	10	110	12	4	Negative
Catechin	No	289.1	Unit/Enh (6490)	202.9	Unit/Enh (6490)	10	110	18	4	Negative
Epicatechin	No	289	Unit/Enh (6490)	244.9	Unit/Enh (6490)	10	110	12	4	Negative
Epicatechin	No	289	Unit/Enh (6490)	202.9	Unit/Enh (6490)	10	110	18	4	Negative
Luteolin	No	284.9	Unit/Enh (6490)	150.7	Unit/Enh (6490)	10	130	26	4	Negative
Luteolin	No	284.9	Unit/Enh (6490)	132.9	Unit/Enh (6490)	10	130	36	4	Negative
Naringenin	No	270.9	Unit/Enh (6490)	150.8	Unit/Enh (6490)	10	90	14	4	Negative
Naringenin	No	270.9	Unit/Enh (6490)	118.8	Unit/Enh (6490)	10	90	28	4	Negative
Apigenin	No	268.9	Unit/Enh (6490)	224.8	Unit/Enh (6490)	10	120	20	4	Negative
Galangin	No	268.9	Unit/Enh (6490)	212.8	Unit/Enh (6490)	10	90	24	4	Negative
Galangin	No	268.9	Unit/Enh (6490)	168.8	Unit/Enh (6490)	10	90	28	4	Negative
Apigenin	No	268.9	Unit/Enh (6490)	116.8	Unit/Enh (6490)	10	120	42	4	Negative
Chrysin	No	252.8	Unit/Enh (6490)	208.8	Unit/Enh (6490)	10	90	22	4	Negative
Chrysin	No	252.8	Unit/Enh (6490)	106.6	Unit/Enh (6490)	10	90	24	4	Negative
Resveratrol	No	226.8	Unit/Enh (6490)	184.8	Unit/Enh (6490)	10	90	18	4	Negative
Resveratrol	No	226.8	Unit/Enh (6490)	142.9	Unit/Enh (6490)	10	90	26	4	Negative
Sinapic Acid	No	222.8	Unit/Enh (6490)	207.8	Unit/Enh (6490)	10	80	8	4	Negative
Sinapic Acid	No	222.8	Unit/Enh (6490)	163.9	Unit/Enh (6490)	10	80	10	4	Negative
Syringic Acid	No	196.9	Unit/Enh (6490)	181.8	Unit/Enh (6490)	10	80	10	4	Negative
Syringic Acid	No	196.9	Unit/Enh (6490)	122.8	Unit/Enh (6490)	10	80	24	4	Negative
Ferulic Acid	No	193	Unit/Enh (6490)	177.9	Unit/Enh (6490)	10	70	10	4	Negative
Ferulic Acid	No	193	Unit/Enh (6490)	134	Unit/Enh (6490)	10	70	14	4	Negative
Quinic Acid	No	190.9	Unit/Enh (6490)	92.9	Unit/Enh (6490)	10	90	22	4	Negative
Quinic Acid	No	190.9	Unit/Enh (6490)	85	Unit/Enh (6490)	10	90	22	4	Negative
Caffeic Acid	No	178.8	Unit/Enh (6490)	134.8	Unit/Enh (6490)	10	80	14	4	Negative
Gallic Acid	No	168.9	Unit/Enh (6490)	124.9	Unit/Enh (6490)	10	80	12	4	Negative
Gallic Acid	No	168.9	Unit/Enh (6490)	79	Unit/Enh (6490)	10	80	24	4	Negative
Vanillic Acid	No	166.9	Unit/Enh (6490)	151.9	Unit/Enh (6490)	10	70	12	4	Negative
Vanillic Acid	No	166.9	Unit/Enh (6490)	122.9	Unit/Enh (6490)	10	70	8	4	Negative
p-Coumaric Acid	No	163	Unit/Enh (6490)	118.9	Unit/Enh (6490)	10	70	14	4	Negative
p-Coumaric Acid	No	163	Unit/Enh (6490)	92.8	Unit/Enh (6490)	10	70	34	4	Negative
Vanillin	No	151	Unit/Enh (6490)	135.8	Unit/Enh (6490)	10	60	10	4	Negative
Vanillin	No	151	Unit/Enh (6490)	91.9	Unit/Enh (6490)	10	60	20	4	Negative
4-OH-Benzoic Acid	No	137	Unit/Enh (6490)	93.1	Unit/Enh (6490)	10	70	14	4	Negative
4-OH-Benzoic Acid	No	137	Unit/Enh (6490)	65.4	Unit/Enh (6490)	10	70	36	4	Negative
Pyrogallol	No	124.9	Unit/Enh (6490)	106.7	Unit/Enh (6490)	10	90	14	4	Negative
Pyrogallol	No	124.9	Unit/Enh (6490)	96.6	Unit/Enh (6490)	10	90	14	4	Negative
Fumaric Acid	No	114.9	Unit/Enh (6490)	71.1	Unit/Enh (6490)	10	50	4	4	Negative
