# Supplementary material for: Multidirectional in silico and in vitro Research for the Pharmaceutical Potential of Fibigia Clypeata (L.) Medik: Phytochemical, Antimicrobial, and Antimyeloma Properties
Source: ChemistryOpen. 2025 Sep 4;14(12):e202500036. doi: 10.1002/open.202500036 (PMC12680575; doi:10.1002/open.202500036)
Supplement: Supplementary file 1 — Supplementary Material [file OPEN-14-e202500036-s001.zip › OPEN-202500036-sup-0001-suppdata-S1/Supporting Information 3.pdf]

Quantitative Analysis Summary Report

**Batch Data Path**	D:\MassHunter\Data\2023\01-Ocak\230130_Phenolic-Compounds\QuantResults\Phenolic_Compunds.batch.bin		
**Analysis Time**	2/1/2023 9:25 AM	**Analyst Name**	LCMS\admin
**Report Time**	2/1/2023 10:19 AM	**Reporter Name**	LCMS\admin
**Last Calib Update**	2/1/2023 9:25 AM	**Batch State**	Processed
**Quant Batch Version**	B.07.01	**Quant Report Version**	B.07.01

Sequence Table

Data File	Acq Method File	Sample Name	Sample Type	Position	Volume	Level
1FM.d	Phenolic_Compounds.m	1FM	Sample	P1-B5	-1.00	
2FS.d	Phenolic_Compounds.m	2FS	Sample	P1-B6	-1.00	
3TM.d	Phenolic_Compounds.m	3TM	Sample	P1-B7	-1.00	
4TS.d	Phenolic_Compounds.m	4TS	Sample	P1-B8	-1.00	

Quantitation Results

Target Compound *Quinic Acid*

Data File	Compound	Sample Type	Response	Final Conc	Unit
1FM.d	Quinic Acid	Sample	258	56.0396	ng/ml
2FS.d	Quinic Acid	Sample	118	2.1988	ng/ml
3TM.d	Quinic Acid	Sample	20391	7811.5591	ng/ml
4TS.d	Quinic Acid	Sample	22918	8784.9784	ng/ml

Target Compound *Fumaric Acid*

Data File	Compound	Sample Type	Response	Final Conc	Unit
1FM.d	Fumaric Acid	Sample	460	245.4418	ng/ml
2FS.d	Fumaric Acid	Sample	6152	3664.9135	ng/ml
3TM.d	Fumaric Acid	Sample	942	535.0279	ng/ml
4TS.d	Fumaric Acid	Sample	1036	591.6005	ng/ml

Target Compound *Gallic Acid*

Data File	Compound	Sample Type	Response	Final Conc	Unit
1FM.d	Gallic Acid	Sample	283	0.0000	ng/ml
2FS.d	Gallic Acid	Sample	8	0.0000	ng/ml
3TM.d	Gallic Acid	Sample	2721	72.0511	ng/ml
4TS.d	Gallic Acid	Sample	7995	254.1032	ng/ml

Target Compound *Pyrogallol*

Data File	Compound	Sample Type	Response	Final Conc	Unit
1FM.d	Pyrogallol	Sample	4	0.0000	ng/ml
2FS.d	Pyrogallol	Sample	1	0.0000	ng/ml
3TM.d	Pyrogallol	Sample	1	0.0000	ng/ml
4TS.d	Pyrogallol	Sample	17	0.0000	ng/ml

Target Compound *Keracyanin Chloride*

Data File	Compound	Sample Type	Response	Final Conc	Unit
1FM.d	Keracyanin Chloride	Sample	807	241.2417	ng/ml
2FS.d	Keracyanin Chloride	Sample	185	55.4636	ng/ml
3TM.d	Keracyanin Chloride	Sample	368	109.9688	ng/ml
4TS.d	Keracyanin Chloride	Sample	20	6.0536	ng/ml

Target Compound *Cyanidin-3-o-glucoside*

Data File	Compound	Sample Type	Response	Final Conc	Unit
1FM.d	Cyanidin-3-o-glucoside	Sample	5387	543.5360	ng/ml
2FS.d	Cyanidin-3-o-glucoside	Sample	3360	339.0656	ng/ml
3TM.d	Cyanidin-3-o-glucoside	Sample	2852	287.7215	ng/ml
4TS.d	Cyanidin-3-o-glucoside	Sample	982	99.0995	ng/ml

Target Compound *Chlorogenic Acid*

Data File	Compound	Sample Type	Response	Final Conc	Unit
1FM.d	Chlorogenic Acid	Sample	1301	5.7660	ng/ml
2FS.d	Chlorogenic Acid	Sample	121	0.0000	ng/ml
3TM.d	Chlorogenic Acid	Sample	355489	13576.7523	ng/ml

Data File	Compound	Sample Type	Response	Final Conc	Unit
4TS.d	Chlorogenic Acid	Sample	26631	976.2811	ng/ml

Target Compound *Catechin*

Data File	Compound	Sample Type	Response	Final Conc	Unit
1FM.d	Catechin	Sample	7	0.0000	ng/ml
2FS.d	Catechin	Sample	2	0.0000	ng/ml
3TM.d	Catechin	Sample	8	0.0000	ng/ml
4TS.d	Catechin	Sample	221	0.0000	ng/ml

Target Compound *Peonidin-3-o-glucoside*

Data File	Compound	Sample Type	Response	Final Conc	Unit
1FM.d	Peonidin-3-o-glucoside	Sample	0	0.0000	ng/ml
2FS.d	Peonidin-3-o-glucoside	Sample	0	0.0000	ng/ml
3TM.d	Peonidin-3-o-glucoside	Sample	0	0.0000	ng/ml
4TS.d	Peonidin-3-o-glucoside	Sample	0	0.0000	ng/ml

Target Compound *4-OH-Benzoic Acid*

Data File	Compound	Sample Type	Response	Final Conc	Unit
1FM.d	4-OH-Benzoic Acid	Sample	1694	0.0000	ng/ml
2FS.d	4-OH-Benzoic Acid	Sample	927	0.0000	ng/ml
3TM.d	4-OH-Benzoic Acid	Sample	429	0.0000	ng/ml
4TS.d	4-OH-Benzoic Acid	Sample	458	0.0000	ng/ml

Target Compound *Epicatechin*

Data File	Compound	Sample Type	Response	Final Conc	Unit
1FM.d	Epicatechin	Sample	3	0.0000	ng/ml
2FS.d	Epicatechin	Sample	9	0.0000	ng/ml
3TM.d	Epicatechin	Sample	7806	644.3273	ng/ml
4TS.d	Epicatechin	Sample	15721	1356.6801	ng/ml

Target Compound *Epigallocatechin Gallate*

Data File	Compound	Sample Type	Response	Final Conc	Unit
1FM.d	Epigallocatechin Gallate	Sample	3	0.0000	ng/ml
2FS.d	Epigallocatechin Gallate	Sample	1	0.0000	ng/ml
3TM.d	Epigallocatechin Gallate	Sample	1	0.0000	ng/ml
4TS.d	Epigallocatechin Gallate	Sample	1	0.0000	ng/ml

Target Compound *Caffeic Acid*

Data File	Compound	Sample Type	Response	Final Conc	Unit
1FM.d	Caffeic Acid	Sample	60	0.0000	ng/ml
2FS.d	Caffeic Acid	Sample	40	0.0000	ng/ml
3TM.d	Caffeic Acid	Sample	3168	0.0000	ng/ml
4TS.d	Caffeic Acid	Sample	757	0.0000	ng/ml

Target Compound *Vanillic Acid*

Data File	Compound	Sample Type	Response	Final Conc	Unit
1FM.d	Vanillic Acid	Sample	211	374.2646	ng/ml
2FS.d	Vanillic Acid	Sample	361	688.4886	ng/ml
3TM.d	Vanillic Acid	Sample	7	0.0000	ng/ml
4TS.d	Vanillic Acid	Sample	23	0.0000	ng/ml

Target Compound *Syringic Acid*

Data File	Compound	Sample Type	Response	Final Conc	Unit
1FM.d	Syringic Acid	Sample	9	0.0000	ng/ml
2FS.d	Syringic Acid	Sample	22	0.0000	ng/ml
3TM.d	Syringic Acid	Sample	5	0.0000	ng/ml
4TS.d	Syringic Acid	Sample	4	0.0000	ng/ml

Target Compound *Vitexin*

Data File	Compound	Sample Type	Response	Final Conc	Unit
1FM.d	Vitexin	Sample	98	0.0000	ng/ml
2FS.d	Vitexin	Sample	43	0.0000	ng/ml
3TM.d	Vitexin	Sample	103132	1410.1860	ng/ml
4TS.d	Vitexin	Sample	69725	929.2669	ng/ml

Target Compound *Naringin*

Data File	Compound	Sample Type	Response	Final Conc	Unit
1FM.d	Naringin	Sample	3	0.0000	ng/ml
2FS.d	Naringin	Sample	7	0.0000	ng/ml
3TM.d	Naringin	Sample	300	3.6679	ng/ml
4TS.d	Naringin	Sample	181	0.0000	ng/ml

Target Compound *Ellagic Acid*

Data File	Compound	Sample Type	Response	Final Conc	Unit
1FM.d	Ellagic Acid	Sample	37	0.0000	ng/ml
2FS.d	Ellagic Acid	Sample	5	0.0000	ng/ml
3TM.d	Ellagic Acid	Sample	7	0.0000	ng/ml
4TS.d	Ellagic Acid	Sample	5	0.0000	ng/ml

Target Compound *Hesperidin*

Data File	Compound	Sample Type	Response	Final Conc	Unit
1FM.d	Hesperidin	Sample	13	0.0000	ng/ml
2FS.d	Hesperidin	Sample	10	0.0000	ng/ml
3TM.d	Hesperidin	Sample	17	0.0000	ng/ml
4TS.d	Hesperidin	Sample	10	0.0000	ng/ml

Target Compound *p-Coumaric Acid*

Data File	Compound	Sample Type	Response	Final Conc	Unit
1FM.d	p-Coumaric Acid	Sample	1850	0.0000	ng/ml
2FS.d	p-Coumaric Acid	Sample	49884	954.4555	ng/ml
3TM.d	p-Coumaric Acid	Sample	725	0.0000	ng/ml
4TS.d	p-Coumaric Acid	Sample	213	0.0000	ng/ml

Target Compound *Sinapic Acid*

Data File	Compound	Sample Type	Response	Final Conc	Unit
1FM.d	Sinapic Acid	Sample	18	0.0000	ng/ml
2FS.d	Sinapic Acid	Sample	15	0.0000	ng/ml
3TM.d	Sinapic Acid	Sample	12	0.0000	ng/ml
4TS.d	Sinapic Acid	Sample	2	0.0000	ng/ml

Target Compound *Taxifolin*

Data File	Compound	Sample Type	Response	Final Conc	Unit
1FM.d	Taxifolin	Sample	29	0.0000	ng/ml
2FS.d	Taxifolin	Sample	6	0.0000	ng/ml
3TM.d	Taxifolin	Sample	74	0.0000	ng/ml
4TS.d	Taxifolin	Sample	46	0.0000	ng/ml

Target Compound *Ferulic Acid*

Data File	Compound	Sample Type	Response	Final Conc	Unit
1FM.d	Ferulic Acid	Sample	1668	344.8477	ng/ml
2FS.d	Ferulic Acid	Sample	7646	1731.0207	ng/ml
3TM.d	Ferulic Acid	Sample	272	21.0421	ng/ml
4TS.d	Ferulic Acid	Sample	119	0.0000	ng/ml

Target Compound *Rosmarinic Acid*

Data File	Compound	Sample Type	Response	Final Conc	Unit
1FM.d	Rosmarinic Acid	Sample	452	127.8775	ng/ml
2FS.d	Rosmarinic Acid	Sample	362	105.7178	ng/ml
3TM.d	Rosmarinic Acid	Sample	1736	442.5273	ng/ml
4TS.d	Rosmarinic Acid	Sample	47	28.6267	ng/ml

Target Compound *Vanillin*

Data File	Compound	Sample Type	Response	Final Conc	Unit
1FM.d	Vanillin	Sample	0	0.0000	ng/ml
2FS.d	Vanillin	Sample	0	0.0000	ng/ml
3TM.d	Vanillin	Sample	0	0.0000	ng/ml
4TS.d	Vanillin	Sample	0	0.0000	ng/ml

Target Compound *Myricetin*

Data File	Compound	Sample Type	Response	Final Conc	Unit
1FM.d	Myricetin	Sample	20	0.0000	ng/ml
2FS.d	Myricetin	Sample	1	0.0000	ng/ml
3TM.d	Myricetin	Sample	3	0.0000	ng/ml
4TS.d	Myricetin	Sample	1	0.0000	ng/ml

Target Compound *Resveratrol*

Data File	Compound	Sample Type	Response	Final Conc	Unit
1FM.d	Resveratrol	Sample	0	0.0000	ng/ml
2FS.d	Resveratrol	Sample	0	0.0000	ng/ml
3TM.d	Resveratrol	Sample	0	0.0000	ng/ml
4TS.d	Resveratrol	Sample	0	0.0000	ng/ml

Target Compound *Luteolin*

Data File	Compound	Sample Type	Response	Final Conc	Unit
1FM.d	Luteolin	Sample	19	0.0000	ng/ml
2FS.d	Luteolin	Sample	64	0.0000	ng/ml
3TM.d	Luteolin	Sample	36008	206.7238	ng/ml
4TS.d	Luteolin	Sample	9310	0.0000	ng/ml

Target Compound *Quercetin*

Data File	Compound	Sample Type	Response	Final Conc	Unit
1FM.d	Quercetin	Sample	611	0.0000	ng/ml
2FS.d	Quercetin	Sample	17	0.0000	ng/ml
3TM.d	Quercetin	Sample	794	0.0000	ng/ml
4TS.d	Quercetin	Sample	293	0.0000	ng/ml

Target Compound *Apigenin*

Data File	Compound	Sample Type	Response	Final Conc	Unit
1FM.d	Apigenin	Sample	2	0.0000	ng/ml
2FS.d	Apigenin	Sample	1	0.0000	ng/ml
3TM.d	Apigenin	Sample	1438	0.0000	ng/ml
4TS.d	Apigenin	Sample	169	0.0000	ng/ml

Target Compound *Naringenin*

Data File	Compound	Sample Type	Response	Final Conc	Unit
1FM.d	Naringenin	Sample	7	0.0000	ng/ml
2FS.d	Naringenin	Sample	0	0.0000	ng/ml
3TM.d	Naringenin	Sample	28	0.0000	ng/ml
4TS.d	Naringenin	Sample	62	0.0000	ng/ml

Target Compound *Isorhamnetin*

Data File	Compound	Sample Type	Response	Final Conc	Unit
1FM.d	Isorhamnetin	Sample	930	0.0000	ng/ml
2FS.d	Isorhamnetin	Sample	8	0.0000	ng/ml
3TM.d	Isorhamnetin	Sample	85	0.0000	ng/ml
4TS.d	Isorhamnetin	Sample	18	0.0000	ng/ml

Target Compound *Chrysin*

Data File	Compound	Sample Type	Response	Final Conc	Unit
1FM.d	Chrysin	Sample	2	0.0000	ng/ml
2FS.d	Chrysin	Sample	3	0.0000	ng/ml

Data File	Compound	Sample Type	Response	Final Conc	Unit
3TM.d	Chrysin	Sample	9	0.0000	ng/ml
4TS.d	Chrysin	Sample	0	0.0000	ng/ml

Target Compound *Galangin*

Data File	Compound	Sample Type	Response	Final Conc	Unit
1FM.d	Galangin	Sample	1	0.0000	ng/ml
2FS.d	Galangin	Sample	2	0.0000	ng/ml
3TM.d	Galangin	Sample	1	0.0000	ng/ml
4TS.d	Galangin	Sample	1	0.0000	ng/ml

Target Compound *Curcumin*

Data File	Compound	Sample Type	Response	Final Conc	Unit
1FM.d	Curcumin	Sample	2	0.0000	ng/ml
2FS.d	Curcumin	Sample	0	0.0000	ng/ml
3TM.d	Curcumin	Sample	2	0.0000	ng/ml
4TS.d	Curcumin	Sample	9	0.0000	ng/ml
